# GSDMD enhances cisplatin-induced apoptosis by promoting the phosphorylation of eIF2α and activating the ER-stress response

**DOI:** 10.1038/s41420-022-00915-8

**Published:** 2022-03-14

**Authors:** Qianyu Zhang, Zixian Huang, Xi Rui, Yan Wang, Yongqiang Wang, Yuwei Zhou, Rui Chen, Yongju Chen, Yuepeng Wang, Shihao Li, Haigang Li, Ximing Shen, Yancan Liang, Yin Zhang, Zhiquan Huang

**Affiliations:** 1 Department of Oral and Maxillofacial Surgery, Sun Yat-sen Memorial Hospital, Sun Yat-sen University, Guangzhou, Guangdong China; 2 Guangdong Provincial Key Laboratory of Malignant Tumor Epigenetics and Gene Regulation, Sun Yat-Sen Memorial Hospital, Sun Yat-Sen University, Guangzhou, China; 3 Medical Research Center, Sun Yat-Sen Memorial Hospital, Sun Yat-Sen University, Guangzhou, China; 4 Hospital of Stomatology, Guanghua School of Stomatology, Guangdong Provincial Key Laboratory of Stomatology, Sun Yat-sen University, Guangzhou, China; 5 Department of Pathology, Sun Yat-sen Memorial Hospital, Sun Yat-sen University, Guangzhou, Guangdong China

**Keywords:** Oral cancer, Chemotherapy

## Abstract

GSDMD is the key effector of pyroptosis, but its non-pyroptosis-related functions have seldom been reported. Here, we report that GSDMD is overexpressed in different types of tumours, including head and neck squamous-cell carcinoma, and it promotes the sensitivity of tumour cells to cisplatin. Unexpectedly, the enhanced cisplatin sensitivity is mediated by apoptosis but not pyroptosis, the well-known function of GSDMD. Furthermore, we found that GSDMD can activate the unfolded protein response by promoting the phosphorylation of eIF2α. Mechanistically, we demonstrated that GSDMD can directly bind to eIF2α and enhance the interaction between eIF2α and its upstream kinase PERK, leading to eIF2α phosphorylation. Consequently, the protein levels of ATF-4 were upregulated, downstream apoptosis-related proteins such as CHOP were activated, and apoptosis was induced. Remarkably, activation of endoplasmic-reticulum (ER) stress induced by GSDMD promotes cell apoptosis during cisplatin chemotherapy, thereby increasing the treatment sensitivity of tumours. Therefore, for the first time, our work reveals an unreported nonpyroptotic function of the classic pyroptosis protein GSDMD: it promotes cell apoptosis during cisplatin chemotherapy by inducing eIF2α phosphorylation and ER stress, which are related to the drug sensitivity of tumours. Our study also indicated that GSDMD might serve as a biomarker for cisplatin sensitivity.

## Introduction

The gasdermin family includes important protein effectors in pyroptosis, a unique type of cell death [1]. After being activated by lipopolysaccharide, viruses or related drugs, gasdermin proteins can be cleaved by different caspases, such as caspase-1 and caspase-4/5, to release the N-terminal functional domain and perforate the cell membrane, causing the cell to swell and die [2–5]. The tumorigenesis process is closely related to various types of stress, including inflammation and bacterial and viral infections, which can trigger pyroptosis. Theoretically, these suicide genes of the gasdermin family should be silenced during tumorigenesis. As expected, gasdermin A, C and E were found to be underexpressed or not expressed in most types of tumours, which was caused by methylation of the promoter or mRNA [6–10]. For example, the expression of GSDME in tumours is significantly lower than that in adjacent tissues [11]. Shao Feng et al. reported that the overexpression of GSDME can cause TNF-α-induced pyroptosis of tumour cells [12]. Moreover, the inflammatory environment and related substances that are ubiquitous in the tumour environment, such as ATP released after cell death, can easily activate the NLRP3 inflammasome pathway and cleave gasdermin D (GSDMD) by activating caspase-1 and cause tumour-cell pyroptosis [13, 14]. However, a previous study and our analysis revealed that the expression of one of the most important members of the gasdermin family, GSDMD, is significantly elevated in different types of cancers [6, 15–17]. The observation that GSDMD is highly expressed in tumours indicates that GSDMD could have functions other than pyroptosis.

In addition to the harsh microenvironment mentioned above, precancerous or cancer cells also encounter different external and internal factors that cause the accumulation of improperly folded proteins, which result in the endoplasmic-reticulum stress (ER stress) [18–21]. In this situation, an adaptive pathway called unfolded protein response (UPR) is activated to address the challenge and help cells to survive [22–24]. UPR mainly triggers three receptors: PKR-like ER-associated protein kinase (PERK), activating transcription factor-6 (ATF-6), and inositol-requiring enzyme-1 (IRE1) [25]. UPR restores cell homoeostasis via several different mechanisms, such as reducing protein translation, increasing chaperone-protein synthesis, promoting ER-folding ability and increasing the expression of ER-related degradation proteins [19, 26]. Increasing studies have revealed the importance of ER stress in tumorigenesis and tumour progression [24]. However, in different stages of tumours and different environments, the effects of ER stress are more complicated than expected [27]. Under some conditions, overactivation can switch the effects of ER stress from adaptive homoeostasis to programmed death, such as apoptosis, but the mechanisms and regulatory molecules in this switch remain to be further explored [28, 29].

Here, we report that GSDMD, previously reported mainly as a pyroptosis effector, is highly expressed in tumours and enhances cisplatin-induced apoptosis. We further demonstrated that GSDMD can activate ER-stress response by promoting the phosphorylation of eIF2α. Mechanistically, we found that GSDMD can enhance the binding of PERK and IF2α, leading to eIF2α phosphorylation. Our work revealed a new function of GSDMD: it promotes the phosphorylation of eIF2α and induces ER stress. When cells were treated with cisplatin, GSDMD-promoted ER stress was overactivated and resulted in apoptosis via the ATF-4-CHOP caspase-3 axis. We also indicated that the silencing of upstream molecules, such as the NLRP3 inflammasome that activates caspase-1 and the cleavage of GSDMD, could be the reason for the absence of pyroptosis. Our study suggests that GSDMD could serve as a biomarker for cisplatin sensitivity.

## Results

### GSDMD is overexpressed in tumours and enhances cisplatin sensitivity

**Fig. 1:**
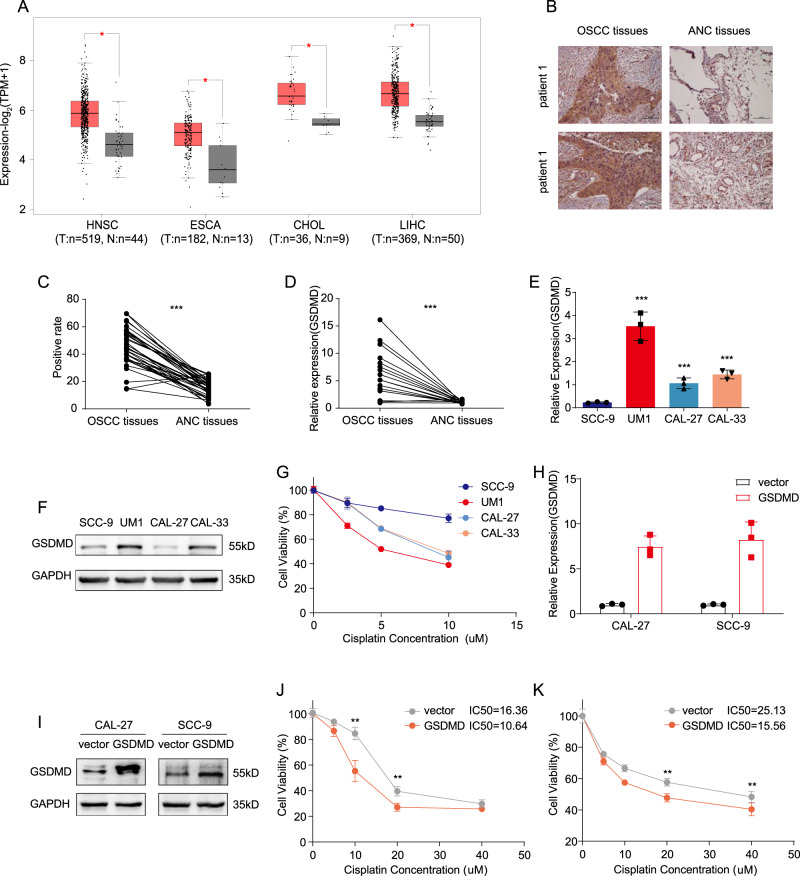
The expression of GSDMD in cancer tissues and paracarcinoma tissues and its effect on cisplatin sensitivity. **A** The TCGA database was assessed to determine the expression of GSDMD in cancer and adjacent cancers. **B** Representative image showed that the expression level of GSDMD in tumour tissues was significantly higher than that in adjacent tissues (upper and lower show the immunohistochemical staining results of paired tissues from two patients; magnification, 100x). **C** The statistical results for **B** (*n* = 36). **D** qPCR showing the expression of GSDMD in tumour tissues and in adjacent normal tissues (*n* = 36). **E** qPCR showing the mRNA levels of GSDMD in multiple cell lines. **F** Western blot showing the protein levels of GSDMD in multiple cell lines. **G** Cell lines with different GSDMD expression were treated with cisplatin at a concentration gradient for 48 h to determine the IC50 of cisplatin in each cell line. **H**, **I** qPCR (**H**) and western blot (**I**) showing the GSDMD expression in Cal-27 and SCC-9 GSDMD-overexpression stable cell line. **J**, **K** A gradient of cisplatin concentrations was used to determine the IC50, the IC50 of control- and GSDMD-overexpressing Cal-27 cells were 16.36 μM and 10.64 μM, respectively (**J**). The IC50 of control and GSDMD-overexpressing SCC-9 cells were 25.13 μM and 15.56 μM, respectively (**K**).

Previous studies have assessed the expression levels of gasdermin-family-related proteins in cancer and adjacent cancers. Due to promoter or mRNA methylation, GSDMA, GSDMC, and GSDME are expressed at low levels or are not in tumours [6, 16, 17]. The expression of GSDMD in tumours remains to be further investigated. Using TCGA data, we statistically compared the expression of GSDMD in cancer tissues and adjacent tissues, and found that, in a variety of tumours, including breast cancer, liver cancer, oesophageal cancer, and head and neck squamous-cell carcinoma, the expression of GSDMD in cancer tissues was significantly higher than that in adjacent tissues (Fig. 1A). At the same time, data based on 36 pairs of clinical samples from Oral squamous-cell carcinoma (OSCC) patients showed the same trend. Data from the immunohistochemical staining of paraffin sections and RT-qPCR of RNA from frozen specimens showed that the expression of GSDMD in tumour tissues was significantly higher than that in adjacent tissues (Fig. 1B, C, D).

The gasdermin family is closely related to the process of cell death, and a previous study conducted by Shao and colleagues [12] showed that overexpression of GSDME resulted in increase of sensitivity to chemotherapeutic agents, including cisplatin, in cancer cells. To explore the association of GSDMD and cisplatin sensitivity, we first analysed the correlation between GSDMD expression and cisplatin-sensitivity chemotherapy in multiple cell lines in vitro. In general, cells with higher GSDMD expression were more sensitive to cisplatin (Fig. 1E, F, G). To further study the roles of GSDMD in cisplatin sensitivity, two cell lines, Cal-27 and SCC-9, which have relatively low expression of GSDMD, were selected to construct overexpressing cell lines and compare the sensitivity of cisplatin chemotherapy. The IC50 results showed that overexpression of GSDMD significantly increased the cisplatin sensitivity of tumour cells (Fig. 1H–K). Reducing the expression of GSDMD with siRNA led to a decrease in cisplatin sensitivity (Fig. S1).

### GSDMD does not increase cisplatin sensitivity via pyroptosis due to the absence of upstream effectors

**Fig. 2:**
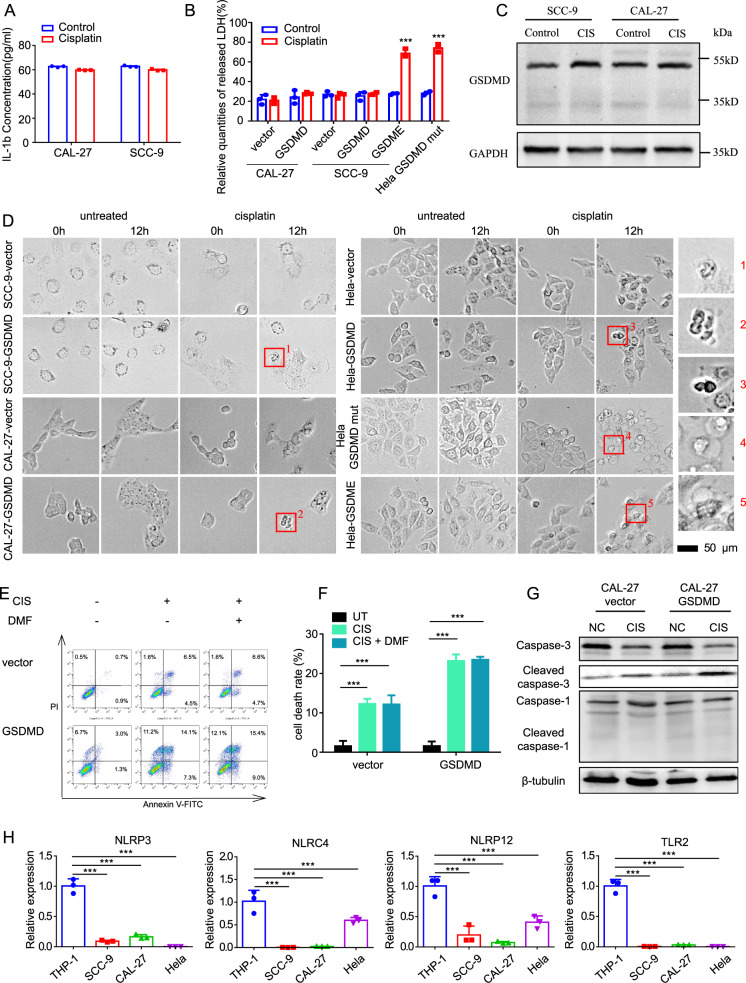
GSDMD increases the cisplatin chemosensitivity of cells through a non-pyroptotic way. **A** ELISA assays showing the release of IL-1β of Cal-27 and SCC-9 cells treated with cisplatin for 24 h. **B** LDH-cytotoxicity experiments showing the LDH release in control and GSDMD-overexpressing cells after cisplatin treatment (24 h). The GSDME-overexpressing cells serve as a positive control. **C** Western blot showing the absence of cleavage of GSDMD in control and GSDMD-overexpressing cells after cisplatin treatment (24 h). **D** Representative images of high-content microscopy live imaging showing the changes of cell morphology after cisplatin treatment. It showed that cells with empty vector and wild-type GSDMD vector did not undergo typical pyroptosis after cisplatin treatment (shown by the blue arrow in the figure), while cells overexpressing GSDME and mutant GSDMD showed typical balloon-like swelling death (shown by the red arrow in the figure). **E** Flow-cytometry assays showing cell death of control and GSDMD-overexpressing Cal-27 cells after cisplatin treatment (24 h). DMF was used as a specific inhibitor of pyrolysis. **F** The statistical-analysis result of flow cytometry assays. **G** Western blot showing the expression and cleavage of caspase-1 and caspase-3. **H** The qPCR showing the expression of NLRP-3, NLRP12 and TLR7 in head and neck squamous- (Cal-27 and SCC-9), cervical cancer (HeLa) and monocyte (THP-1) cell lines.

As mentioned above, GSDMD is the key effector of pyroptosis. To investigate the reason for increased cisplatin sensitivity, we first measured the release of IL-1β and TNF-α, which are widely used markers of pyroptosis. Surprisingly, neither of these markers was significantly elevated (Fig. 2A and Fig. S2) in cells that stalely expressed GSDMD. We further detected the release of LDH, and found that cisplatin resulted in no significant increase in LDH in either control or GSDMD-overexpressing cells.

However, when overexpressed GSDME, which has been reported [12] to induce pyroptosis upon cisplatin treatment, a significant increase in LDH was observed (Fig. 2B). Cleavage of the gasdermin family is the gold standard of pyroptosis at the molecular level; western blotting was next performed to detect whether GSDMD was cleaved. As shown in Fig. 2C, no obvious cleavage of GSDMD was observed upon cisplatin treatment.

GSDMD can be cleaved by caspase-1, which is activated by the inflammasomes, while GSDME is cleaved by caspase-3. GSDME can switch caspase-3-mediated apoptosis-induced chemotherapy drugs to pyroptosis [12]. To further explore the reason of the absence of pyroptosis in GSDMD-expressing cells upon cisplatin treatment, we mutated the original caspase-1 cleavage sequence (FLTD) of GSDMD into the caspase-3 cleavage sequence DEVD (from cleavage site of GSDME) as previously reported [12]. Unlike wild-type GSDMD, overexpression of mutated GSDMD results in dramatic increase of LDH release upon cisplatin treatment. The degree of increased LDH by mutated GSDMD was similar as overexpression of GSDME in Hela, which serves as a positive control for cisplatin-induced pyroptosis. These data indicated that overexpressed GSDMD does not induce pyroptosis in cancer cells, which could be caused by failure to activate inflammatory caspase-1 and cleave off GSDMD.

To confirm that the cell-death type was not pyroptosis, we conducted live-image observation of cell morphology after cisplatin treatment. Regardless of whether GSDMD was overexpressed, no obvious cell swelling was observed, indicating that pyroptosis was not induced. While using both GSDME and GSDMDmut overexpression, stably transfected cell lines undergoing pyroptosis caused by cisplatin-activated cleavage of GSDME by caspase-3 were used. The “balloon-like” swelling type of cell death observed in the positive controls did not appear in the experimental group (Fig. 2D and Fig. S3).

We further used the GSDMD-cleavage inhibitor dimethyl fumarate (DMF) for study. If cell death is caused by GSDMD-cleavage-mediated pyroptosis, DMF should significantly reduce the number of dead cells. Flow cytometry showed that GSDMD overexpression resulted in a significant increase in cell death upon cisplatin treatment, and DMF did not attenuate the cell death caused by GSDMD overexpression (Fig. 2E, F). These data demonstrated that GSDMD promotes cisplatin sensitivity independent of pyroptosis.

To investigate the reason for the absence of pyroptosis in GSDMD-expressing cells upon cisplatin treatment, we assessed the upstream effector of GSDMD, which activates caspases responsible for the cleavage of GSDMD, by qRT-PCR and western blot. Generally, pattern-recognition receptors recognise pathogen-associated molecular patterns and damage-associated molecular patterns and then activate caspase-1 to induce GSDMD-mediated pyroptosis. Pattern-recognition receptors related to pyrolysis mainly include Toll-like receptors (TLRs) and Nod-like receptors (NLRs). Among these receptors, NLRP3, TLR2, etc., participate in the formation of inflammasomes and play important roles in pyroptosis [1]. We found that the expression of NLRP3 in tumour cells was significantly lower than that in normal cells (Fig. S4). A variety of pattern-recognition receptors are silenced in tumour cells compared with THP-1 cells (Fig. 2H and Fig. S5), which is considered to be a classic pyroptosis-research model. In THP-1 cells, GSDMD was successfully activated to cause pyroptosis. As known, caspase-1 is a key protein in GSDMD-dependent pyroptosis, and caspase-3 is a key protein in the process of apoptosis. Therefore, we detected the cleavage of caspase-1 and caspase-3 under cisplatin-chemotherapy treatment by western blotting. The results showed that the cleaved caspase-1 level was not significantly different between the cisplatin-treatment group and the -untreated group. However, the cleaved caspase-3 level was increased in the cisplatin-treatment group compared with the other groups (Fig. 2G). These data indicated that the upstream effector of GSDMD and the cleavage machine might not function due to low expression; thus, caspase-1 cannot be activated to cleave GSDMD to release the N-terminus and induce pyroptosis.

### GSDMD promotes eIF2α phosphorylation by enhancing the interaction between PERK and eIF2α

**Fig. 3:**
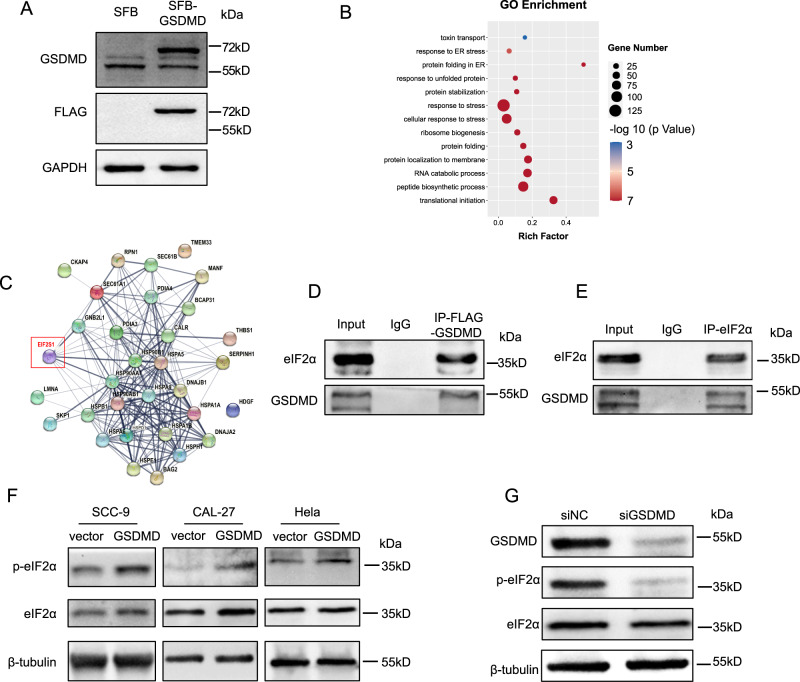
Analysis and verification of GSDMD-interacting proteins. **A** Western blot was used to verify the successful construction of GSDMD-overexpressing cell line. **B** Gene ontology (GO) analysis of GSDMD-interacting proteins identified by co-IP followed by mass spectrometry. **C** Network analysis of GSDMD interacting proteins that involved in ER-stress response. **D** Western blot detecting eIF2α and GSDMD following immunoprecipitation of flag-GSDMD. **E** Western blot detecting eIF2α and GSDMD following immunoprecipitation of eIF2α. **F** Western blot showing the level of phosphorylated eIF2α in the control and GSDMD-overexpression cells. **G** Western blot showing the level of phosphorylated eIF2α when knocking down GSDMD by siRNA.

Functional and mechanistic studies of GSDMD have mainly focused on pyroptosis. To explore why GSDMD promotes cisplatin sensitivity, we systemically identified GSDMD-interacting proteins. We first constructed a cell line with stable overexpression of SFB-tagged GSDMD (Fig. 3A) and then conducted co-IP of GSDMD and mass spectrometry analysis. The proteins that interacted with GSDMD were mainly enriched in protein-translation and ER-stress-related processes (Fig. 3B, C and Fig. S6). Detailed analysis showed that there was a direct interaction between GSDMD and eIF2α, as further confirmed by IP of GSDMD and eIF2α and subsequent western blotting (Fig. 3D, E).

**Fig. 4:**
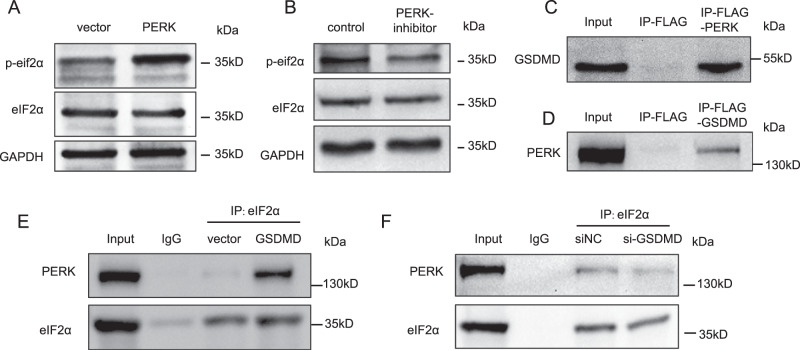
GSDMD promotes eIF2α phosphorylation by enhancing the binding of PERK to eIF2α. **A** Western blot showing the level of phosphorylated eIF2α when overexpressed of PERK or (**B**) treated with PERK inhibitor (GSK2606414, 1 μM, 24 h) in GSDMD-overexpressing cells. **C** Western blot detecting GSDMD following immunoprecipitation of flag-PERK. **D** Western blot detecting PERK following immunoprecipitation of GSDMD. **E**, **F** Western blot following immunoprecipitation showing the change of strength of the binding between GSDMD and PERK when overexpressing (**E**) or knocking down GSDMD (**F**).

eIF2α is an important regulator of the translation process and ER stress. When phosphorylated, it induces global translation inhibition and selectively activates the translation of some downstream genes, such ATF-4. Translation inhibition allows cells to degrade unfolded proteins. To further investigate the consequence of the GSDMD–eIF2α interaction, we detected the phosphorylation level of eIF2α and found that overexpression of GSDMD dramatically increased the level of phosphorylated eIF2α (Fig. 3F). Consistently, knocking down GSDMD significantly reduced the phosphorylation level of eIF2α (Fig. 3G). Moreover, the increase in the phosphorylation level of eIF2α induced by GSDMD was attenuated by the addition of a PERK inhibitor (Fig. 4A, B), indicating that GSDMD-enhanced eIF2α phosphorylation depends on PERK. PERK is the kinase that mediates the phosphorylation of eIF2α, and we speculated that GSDMD could enhance the interaction between PERK and eIF2α. To verify this assumption, we first detected the interaction between GSDMD and PERK. The IP blot of flag-tagged PERK showed a clear GSDMD band, and the IP blot of GSDMD also showed a clear PERK band (Fig. 4C, D). Then, we conducted Co-IP assays in GSDMD-overexpressing and GSDMD-knockdown cell lines. We found that, upon overexpression of GSDMD, the interaction between PERK and eIF2α was significantly increased (Fig. 4E). When GSDMD was silenced, the binding of PERK and eIF2α decreased (Fig. 4F). These data revealed that GSDMD promotes eIF2α phosphorylation by enhancing the interaction between PERK and eIF2α. In addition, to exclude that ER stress is caused by overexpressed GSDMD protein overwhelming the cell protein-expression system, we used GSDME-overexpressing cal-27 cells as controls and detected the phosphorylation level of eIF2α in two stable overexpressing cell lines. The results showed that GSDME overexpression had no significant effect on the phosphorylation level of eIF2α (Fig. S7).

### GSDMD can activate the ER-stress response

**Fig. 5:**
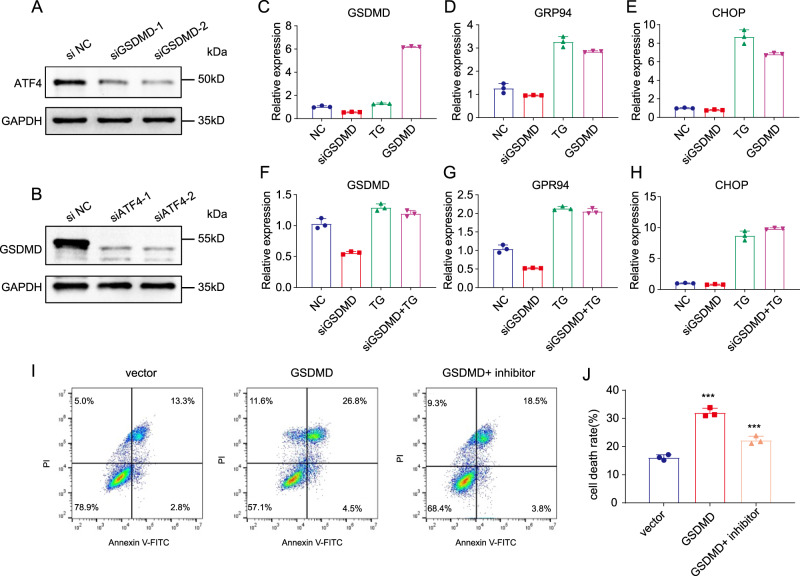
GSDMD increases eIF2α phosphorylation and activates many downstream ER stresses. **A** Western blot showing the expression of GSDMD when knocking down ATF-4. **B** Western blot showing the expression of ATF4 when knocking down GSDMD. **C**–**E** After GSDMD siRNA, TG or GSDMD-overexpression treatment, qPCR was used to detect the change of mRNA level of GSDMD (**C**), ER-stress downstream genes GRP94 (**D**) and CHOP (**E**) in Cal-27 cells. **F**–**H** After GSDMD siRNA, TG or TG + siRNA treatment, qPCR was used to detect the change of mRNA level of GSDMD (**F**), ER-stress downstream genes GRP94 (**G**) and CHOP (**H**) in Cal-27 GSDMD stable overexpression cells. (**I**) Flow cytometry to detect cell death after treatment with cisplatin (48 h), in control cells, GSDMD-overexpression cells and GSDMD-overexpression cells with PERK-inhibitor (GSK2606414, 1 μM, 48 h) treatment. (**J**). The results of statistical analysis of (**I**).

Upon ER stress and eIF2α phosphorylation, the transcription factor ATF-4 is selectively activated by inhibition of its upstream open reading frame (uORF) translation and promotion of its ORF translation. To investigate this process, we detected the expression of ATF-4 by western blotting. Silencing of GSDMD resulted in a significant decrease in ATF-4 at the protein level (Fig. 5A). Interestingly, we also observed a significant decrease in GSDMD when ATF-4 was silenced using two individual siRNAs, indicating that GSDMD might form a positive-feedback loop with eIF2α–ATF-4 (Fig. 5B). We further measured the downstream genes of ATF-4 in GSDMD-overexpressing and GSDMD-knockdown cells since these genes are important effectors of ER stress. Thapsigargin (TG), which induces ER stress, was used as a positive control. The results showed that the expression of multiple ER-stress-related genes, including GRP94 and CHOP, was significantly elevated in GSDMD-overexpressing cells (Fig. 5C–E). Knockdown of GSDMD reduced the expression of genes downstream of ER stress (Fig. 5F–H). These data demonstrated that GSDMD can promote the downstream genes of ER stress.

We then further investigated the relationship between GSDMD and sensitivity to chemotherapeutic drugs. Flow-cytometry detection of apoptosis showed that, upon cisplatin treatment, the apoptosis rate of the GSDMD-overexpression group was significantly higher than that of the control group. Moreover, the PERK inhibitor partially abolished the increase in the apoptosis rate caused by the overexpression of GSDMD (Fig. 5I, J). Western blot analysis also showed that cisplatin increased the eIF2α phosphorylation level and that PERK inhibitors reversed this process (Fig. S8). In addition to PERK inhibitors, we also used PERK-targeted siRNA for knockdown. The results were similar to the use of inhibitors, which could dramatically reduce the apoptosis caused by cisplatin treatment (Fig. S9).

### GSDMD induces apoptosis through ER stress

**Fig. 6:**
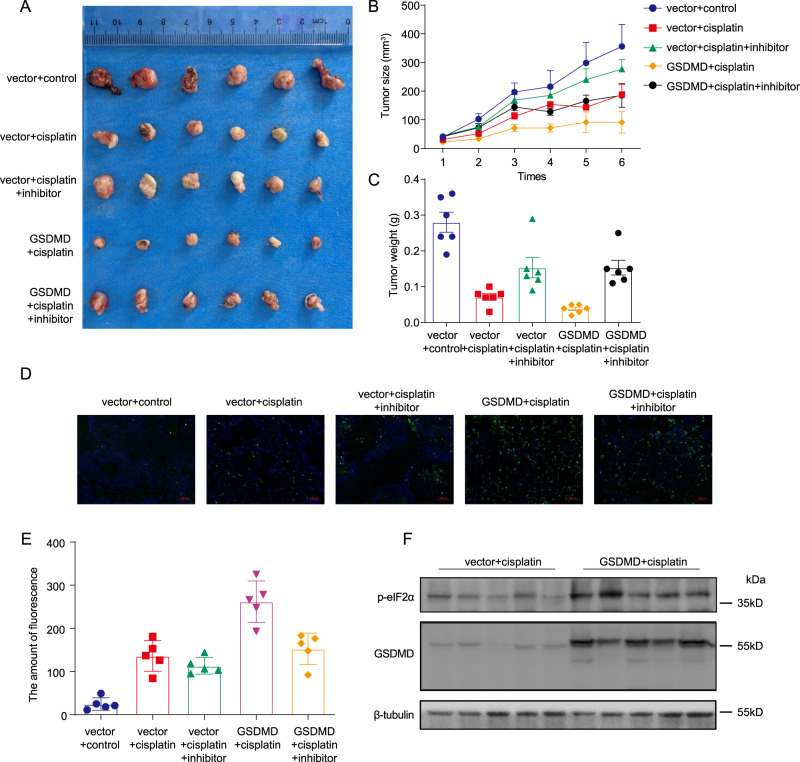
The chemotherapy model of transplanted tumours in nude mice verified the promoting effect of GSDMD on cisplatin sensitivity. **A** Xenograft tumour of empty vector or GSDMD-overexpression Cal-27 cells with different treatment, the representative image of tumours at the endpoint. **B** Growth curve of nude mice from the time of tumour formation to the end of cisplatin chemotherapy. **C** The weight statistics for the tumours with different treatment. **D** TUNEL staining (green fluorescence) was applied to detect cell apoptosis in transplanted tumours. DAPI staining was used to indicate the cell nucleus. **E** Statistical analysis of tunel staining. **F** The tumours were homogenised and the protein of GSDMD and phosphorylated eIF2α was detected by western blot.

To explore the role of GSDMD in vivo, we next constructed a nude mouse xenograft tumour model using GSDMD-overexpressing and negative conrol cells, respectively. Cisplatin and a combination of cisplatin and PERK inhibitor were used to treat these tumours, and the tumour volumes were measured (Fig. 6A). The results showed that cisplatin chemotherapy could significantly inhibit the growth of transplanted tumours, as reflected by the small size and light weight of the tumours. This inhibitory effect was more obvious in the GSDMD-overexpression group than in the control group. The use of a PERK inhibitor reduced the cisplatin-induced decreases in the volume and weight of transplanted tumours in both the GSDMD-overexpression group and the control group (Fig. 6B, C). The use of PERK inhibitors also reduced the increase in eIF2α phosphorylation caused by GSDMD (Fig. S10). TUNEL staining was used to detect cell apoptosis in paraffin sections. The results showed that the proportion of cells with green fluorescence in the GSDMD-overexpression group after cisplatin chemotherapy was significantly higher than that in the control group. The use of PERK inhibitors in the GSDMD-overexpression group partially reduced the proportion of cells with green fluorescence (Fig. 6D, E). However, PERK inhibitors had no significant effect on green fluorescence in the control group. Western blotting of transplanted tumours showed that the phosphorylation level of eIF2α was higher in transplanted tumours overexpressing GSDMD than in control tumours (Fig. 6F).

### GSDMD induces apoptosis through ER stress during cisplatin chemotherapy of tongue squamous-cell carcinoma

**Fig. 7:**
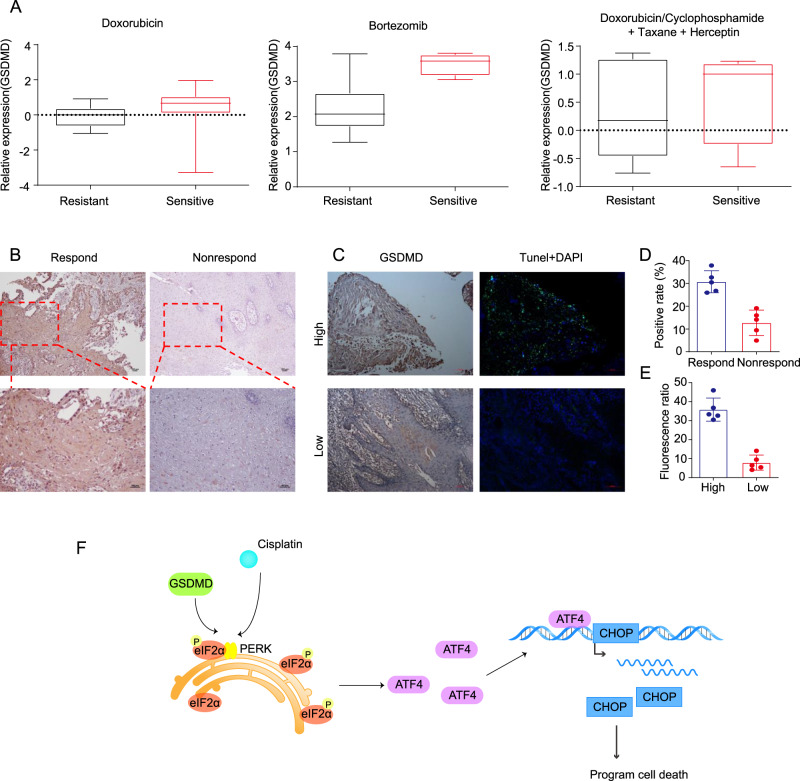
GSDMD expression and apoptosis detection in patient samples after neoadjuvant chemotherapy. **A** Data from the ONCOMINE database showed that patients with high expression of GSDMD are more sensitive to drugs, including doxorubicin, bortezomib and cisplatin, than those with low expression. **B** Representative pictures show that samples from patients with a good response to neoadjuvant chemotherapy have higher expression of GSDMD than those with a poor response. **C** TUNEL staining and immunohistochemical staining of serial sections was performed, DAPI staining was used to indicate the cell nucleus. **D** The statistical results of the positive rate of GSDMD immunohistochemical staining in neoadjuvant chemotherapy-patient samples showed that patients with a good response had higher GSDMD expression (*n* = 10). **E** Statistics show that the group with higher expression of GSDMD has higher green fluorescence. **F** Proposed working model showing that GSDMD can enhance the intercalation between PERK and eIF2α, promote phosphorylation of eIF2α and thus increase ER-stress response and cisplatin sensitivity.

To further investigate the relationship between GSDMD and sensitivity to chemotherapeutic drugs in patients, we first conducted an analysis of the Oncomine database. We found that, for a variety of tumours, including breast cancer, patients with high expression of GSDMD had a better response to chemotherapy regimens containing platinum drugs than patients with low expression (Fig. 7A). We also detected the expression of GSDMD in patients treated with neoadjuvant chemotherapy. With the 50% tumour-residual rate as the boundary between the response and non-response groups, 10 specimens from surgery after neoadjuvant chemotherapy were analysed. Immunohistochemical staining showed that the expression of GSDMD in the response group was significantly higher than that in the non-response group (Fig. 7B, D). TUNEL staining and immunohistochemical staining were performed on serial sections. In areas with high GSDMD expression, TUNEL staining revealed a large proportion of cells with green fluorescence, while in areas with low GSDMD expression, TUNEL staining revealed a small proportion of cells with green fluorescence (Fig. 7C, E).

## Discussion

In summary, we proved a new function of the GSDMD protein in cells: it promotes the phosphorylation of eIF2α to induce ER-stress response and enhance sensitivity to cisplatin chemotherapy. As the main functional protein of pyroptosis, GSDMD has seldom been reported to have biological functions, except in pyroptosis. In this study, we found that GSDMD can increase the binding of eIF2α and PERK and promote the phosphorylation of eIF2α by binding to eIF2α. As a result, the expression of CHOP increases, driving cell death through ATF-4-induced apoptosis. The results revealed that GSDMD can increase the sensitivity of tumours to chemotherapy via ER stress instead of pyroptosis (Fig. 7F).

Generally, GSDMD is mainly responsible for inducing cell pyroptosis [30]. Related studies of the tumour microenvironment have confirmed that, after pyroptosis occurs, it can activate autoimmunity to eliminate tumours. A variety of cytokines, including IL-1β and TNF-α, are released during pyroptosis and can promote the body’s antitumour effect [31, 32]. The difference is that, after apoptosis occurs, cells are cleared by macrophage phagocytosis, and the impact on other cells is relatively small. In this study, it was found that multiple pattern-recognition receptors are underexpressed in tumour cells, making it difficult to activate pyroptosis in tumour cells even if GSDMD is highly expressed. Moreover, high expression of GSDMD activates the apoptotic process through the ER-stress pathway and might eliminate the cells that persist after chemotherapy. This fact might explain why tumour cells avoid pyroptosis during chemotherapy and avoid attack by the host immune system.

The relationship between ER stress and tumours is complicated [25]. ER-stress response promotes the folding function of the ER and removes abnormally synthesised proteins from the cell. ER-stress response reduces the level of protein translation, and the cell enters a state similar to dormancy, thereby maintaining the stability of the intracellular environment [28]. In addition, chronic persistent ER stress has been confirmed to be related to multidrug resistance in cells [33–35]. Conversely, when strong ER stress occurs, cells cannot survive. After ER stress occurs, these nonviable cells are eliminated through apoptosis [36, 37]. Multiple outcomes of ER stress, including intracellular autophagy, decreased translation and apoptosis, have been reported [33]. Previous studies revealed that the intensity of ER stress determines the outcome of cells. It has been reported that the expression of QRICH1, a key transcription factor, determines whether ER stress ends in apoptosis [28]. In our research, the expression of GSDMD was affected by the expression of ATF-4, while the activity of ATF-4 was regulated by GSDMD. This positive mutual regulation could drive cells with high expression of GSDMD to be more inclined to undergo ATF-4–CHOP-related apoptosis as the final outcome of ER stress. Taking together, our research indicates that GSDMD promotes ER stress, enabling tumour cells to clear misfolded and unfolded proteins faster in an inflammatory environment (Fig. S11), supporting the maintenance of cell homoeostasis and providing a survival advantage. However, when chemotherapy is applied as a new stimulus, this survival advantage can be subverted, leading to the death of tumour cells. ER stress activates ATF-4–CHOP-related apoptosis pathways [38]. GSDMD promotes ER stress and could confer a survival advantage in an inflammatory environment, but when cisplatin chemotherapy is introduced as a new stimulus, cell apoptosis can occur.

Chemotherapy insensitivity in tumours is an important reason why it is difficult to improve the prognosis of many tumours. Therefore, improving the effects of existing drugs has always been an important direction for research. We found that GSDMD can promote the binding of PERK to eIF2α and increase the phosphorylation of eIF2α. In the course of cisplatin chemotherapy, GSDMD induces cell apoptosis by promoting eIF2α phosphorylation to activate ER stress and increases the sensitivity of tumours to cisplatin in a nonpyrolytic manner. These findings indicate that GSDMD could be used as an indicator of drug sensitivity in the future. At the same time, GSDMD and eIF2α, as proteins of pyroptosis and endoplasmic-reticulum stress, were shown to interact and promote the phosphorylation of eIF2α in our study. This finding could be the basis for further research on the relationship between ER stress and pyroptosis.

## Materials and methods

### Cell culture

Cell lines (Cal-27, SCC-9, CAL-33, HSC-3, HeLa and HEK-293T) were purchased from the American Type Tissue Culture Collection (ATCC) and identified by short tandem repeat (STR) typing; Cal-27, CAL-33, HSC-3, HeLa and 293 T cells were cultured in Dulbecco’s modified Eagle’s medium (DMEM) supplemented with 10% foetal bovine serum. SCC-9 cells were maintained in Ham’s F-12 nutrient medium supplemented with 10% foetal bovine serum. The Petri dishes containing cells were placed in a constant-temperature incubator at 37 °C and 5% CO_2_ with saturated humidity. The cells grew adherently, and when the cells grew to 80% confluence, 0.25% trypsin digestion was used for passaging.

### Reagents

A PERK inhibitor (GSK2606414, GlaxoSmithKline) was dissolved in absolute ethanol at a concentration of 10 mM for preservation and stored at −80 °C for later use [39, 40].

Thapsigargin (TG, Merck Millipore) was used as an ER-stress activator. It was prepared as a 10 mM preservation solution and stored in a refrigerator at −80 °C for later use.

The cisplatin stock solution at 30 mg/6 mL (S31072, Yuan Ye) was directly diluted with the culture medium to achieve final doses of 0 μM, 2.5 μM, 5 μM, 10 μM, 20 μM, 40 μM, 80 μM and 160 μM cisplatin to assess cell resistance.

### Drug-cytotoxicity detection

Oral squamous-cell carcinoma (OSCC) cells were plated in a 96-well plate at 10,000 cells/well, and after incubating for 24 h, the corresponding cisplatin concentration was configured for treatment according to the aforementioned medication regimen. After 48 h, Cell Counting Kit-8 (CCK-8) assays were used to determine the relative absorbance of the cells, and a conversion formula was used to calculate the half-maximal inhibitory concentration (IC50) of cisplatin in the cells as follows: IC50 = lg-1[Xm-i(ΣP-0.5)] (where Xm is the logarithmic value of the designed maximum concentration, i is the logarithmic value of each concentration ratio, ΣP is the growth-inhibition rate of each group and 0.5 is the empirical constant).

The cell-inhibition rate was calculated as follows: cell-proliferation inhibition rate = (control-group OD value-experimental-group OD value)/control-group OD value×100%

### Flow cytometry

The cells were seeded in a 6-well plate at 300,000 cells/well; after 12 h of incubation, the cells were processed according to the experimental requirements. The supernatant was collected, and the cells were washed with PBS, trypsinized and resuspended in a 15-mL centrifuge tube and centrifuged. After washing with 2 mL of PBS twice, the cells in the pellet were stained with annexin V-FITC/PI according to the instructions. A Becton Dickinson FACScan flow cytometer was used to analyse the stained cells, and FlowJo software was used to process the data.

### Cytotoxicity determination (LDH method)

The cells were seeded in a 96-well plate at 4000 cells/well. Each treatment group had 3 replicate wells. The cells were cultured in a constant-temperature incubator at 37 °C with 5% CO_2_ and saturated humidity for 24 h; the cells were treated according to the experimental requirements. The CytoTox 96 Non-Radioactive Cytotoxicity Assay Kit (#G1781, Promega) was applied to detect cellular LDH release at a specific time point and to calculate the cytotoxicity of the processed drug following the manufacturer’s instructions.

### Plasmid construction and cell transfection

#### Plasmid design

The design and synthesis of GSDMD-overexpression lentiviral vectors were completed by Shandong Weizhen Biotech, and pCDH was used as the overexpression-vector backbone.

A mutant GSDMD plasmid with the caspase-1 cleavage sequence of TTCCTGACAGAT (FLTD) in hGSDMD changed to the caspase-3 cleavage sequence of GATGAAGTGGAT (DEVD) was constructed by Guangzhou IGE Biotechnology Inc Ltd as previously reported [12], flag tags were added at the N-terminus of both wild-type and mutant GSDMD. The pCDH vector was the backbone.

### Virus production and infection and stable cell-line transfection

The GSDMD-overexpression vector and the lentiviral-packaging plasmids pMD2.G (#12259, Addgene) and psPAX2 (#12260, Addgene) were cotransfected into 293 T cells at a ratio of 3:1:2. The supernatant was extracted after 48 h and filtered with a 0.22-μm filter to obtain the lentivirus-infection solution.

Twenty-four hours before transfection, 5000 cells/well of the transfected OSCC cells were plated into a 24-well plate. For transfection, 1 mL of lentiviral-infection solution and 1 μL of polybrene were added into cells in a 24-well plate. The cells were incubated for 36 h. Then, the supernatant was removed, the cells were washed twice with PBS and puromycin (5 μg/mL) was added to select the cells.

After the cell line was constructed, qPCR and western blotting were used to determine whether mRNA and protein expression were promoted or inhibited.

### RNA interference

siRNA was designed and produced by GenePharma Co. (Shanghai, China). For siRNA transfection, cells (3 × 10^5^ cells/well) were seeded in a 6-well plate and incubated overnight, and then 50 nM small-interfering RNA and Lipofectamine® RNAiMAX (Invitrogen, #13778-150) were added for transfection. After 6 h, the cells were cultured with fresh complete culture medium.

### mRNA-expression analysis

TRIzol reagent (Takara) was used to extract total RNA according to the manufacturer’s instructions, PrimeScript™ RT Master Mix (Takara) was added and the RNA was reverse-transcribed into cDNA and analysed by ABI 9700 Real-Time PCR instrument (ABI, USA). Expression of each gene was normalised to GAPDH as internal reference, and quantified using the 2^−ΔΔ^ (ct) method, and the following primers were used (Table 1).

**Table 1:** Primer sequence.

Gene name	Forward primer (5′–3′)	Reverse primer (3′–5′)
GAPDH	GAGTCAACGGATTTGGTCGT	GACAAGCTTCCCGTTCTCAG
GSDMD	TCTGCCCTCAACACTTCTGG	TGCAGCCACAAATAACTCAGC
ATF4	GTTCTCCAGCGACAAGGCTA	ATCCTGCTTGCTGTTGTTGG
CHOP	AGAACCAGGAAACGGAAACAGA	TCTCCTTCATGCGCTGCTTT
XBP-1	TGGCCGGGTCTGCTGAGTCCG	ATCCATGGGGAGATGTTCTGG
NLRP3	GATCTTCGCTGCGATCAACAG	CGTGCATTATCTGAACCCCAC
NLRP1	GCAGTGCTAATGCCCTGGAT	GAGCTTGGTAGAGGAGTGAGG
NLRP2	TGGCCTGGAGATAGCAAAGAG	CACCACCGTGTATGAGAAGGG
NLRP12	GGGGCTTGTCAGGAGATGG	AGTCCCTGGCATAGTAACCTC
NLRC4	TGCCCAGAAATCGAAGCCC	GGCACCAAACTGCCGTATG
tlr2	ATCCTCCAATCAGGCTTCTCT	GGACAGGTCAAGGCTTTTTACA
tlr4	AGACCTGTCCCTGAACCCTAT	CGATGGACTTCTAAACCAGCCA
tlr7	TCGTGGACTGCACAGACAAG	GGTATGTGGTTAATGGTGAGGGT
tlr9	CTGCCTTCCTACCCTGTGAG	GGATGCGGTTGGAGGACAA
Aim2	TCAAGCTGAAATGAGTCCTGC	CTTGGGTCTCAAACGTGAAGG

### Western blotting

After washing the cells with PBS 2 times, lysis buffer was added to lyse the OSCC cells at 4 °C for 30 min. Cell lysates were collected in a 1.5-mL tube and centrifuged at 4 °C at 13,000 rpm for 20 min. The supernatants were collected and the protein quantification was detected using the BCA method, and then diluted and mixed with loading buffer and heated at 95 °C for 5 min for protein denaturation.

The polyacrylamide gel (SDS-PAGE gel) was prepared according to the instructions of the Biyuntian Polyacrylamide Gel Kit, electrophoresis was performed (90 V, 1.3 h) and then the protein was transferred to a PVDF membrane (Immobilon-P transfer membrane) (250 mA, 70 min).

The PVDF membrane was incubated with TBST containing 5% skim milk at room temperature for 1 h for protein blocking; the membrane was washed with TBST 3 times for 5 min each time and incubated with primary antibodies (GSDMD (Abcam, 219800), eIF2α (CST, #5324), GAPDH (CST, #5174) and P-eIF2α (CST, #3398) at 4 °C) for 16 h (overnight); and the next day, after washing 3 times with TBST, the corresponding secondary antibody was added and incubated at room temperature for 2 h. Then, chemiluminescence, photographing, and gel-image analysis were performed.

### High-content cell-imaging system

Cells were seeded in 96-well plates at 15,000/well and incubated overnight. Then, they were treated with cisplatin or TG as previously described. Images were obtained with an ImageXpress Microconfocal High-Content Imaging System every 5 min for 24 h to record the process of cell death. Live-cell imaging was performed in an Okolab Cage Incubator with a constant temperature of 37 °C with 5% CO_2_ and constant humidity. All of the imaging data represent at least three random sites, and the images were processed using the ImageJ software program.

### Enzyme-linked immunosorbent assay (ELISA)

OSCC cells were seeded in a 96-well plate (5000 cells/well) and incubated overnight. Then, the cells were treated with the specified concentration of cisplatin or TG for 24 h, and the supernatant medium was collected. IL-1β, TNF-α and other secretory factors were detected according to the instructions of the ELISA kit.

### Co-immunoprecipitation (Co-IP)

Cell samples were plated in 10-cm cell culture dishes at a cell density of approximately 90% for experimentation. NETN buffer was mixed with protein-phosphatase inhibitor and used as a cell lysate; 1 mL of cell lysate was added to each dish, and the cells were lysed on ice for 20 min. After centrifugation at 4 °C and 13,000 rpm for 15 min, the supernatant was collected for Co-IP.

The unconjugated antibody (endogenous antibody) (1 µg) was added to the lysate and incubated at 4 °C for 1 h; before the incubation, the protein-G magnetic beads were washed twice with NETN buffer, and then the magnetic beads were removed and added to the lysis buffer for incubation overnight at 4 °C on a rotating shaker. The agarose beads coupled with S-protein were washed twice with NETN buffer and incubated with the coupled antibody (exogenous antibody), and then sugar beads were added to the protein-lysis buffer for overnight incubation at 4 °C on a rotating shaker.

For co-immunoprecipitated protein extraction and western blotting, the next day, the magnetic/sugar beads were washed with NETN 5 times. Then, 50 µL of NETN + loading buffer were added and incubated at 95 °C for 5 min for protein denaturation; the supernatant was aspirated, and western blot detection was performed.

### Mass spectrometry

For protein-profile analysis, the protein sample was incubated overnight in the previous step.

For protein elution, the beads were washed 5 times with 100 μL of NH_4_HCO_3_ (100 mM) and centrifuged at 1000 rpm for 1 min; then, the supernatant was removed, 100 μL of protein-lysis buffer (10 mM DTT/100 mM NH_4_HCO_3_ solution) was added and the mixture was centrifuged at 56 °C with shaking for 30 min. After cooling to room temperature, the samples were centrifuged at 1300 rpm to remove the supernatant. Next, 100 μL of 100 mM iodoacetamide (dissolved in 100 mM NH_4_HCO_3_) was added; the samples were shaken at room temperature for 30 min and centrifuged to remove the supernatant. The beads were washed once with 100 μL of NH_4_HCO_3_ (100 mM) and centrifuged at 1000 rpm for 1 min, and the supernatant was removed. The beads were resuspended in 100 μL of NH_4_HCO_3_ (100 mM), 1 µg of trypsin was added at 37 °C and the mixture was shaken at 1000 rpm for 13 h. After adding 0.4 μL of 10% trifluoroacetic acid (TFP) to terminate the digestion, the supernatant was pipetted into a new EP tube, and the protein elution was complete.

After the protein was concentrated and redissolved, it was tested on a computer to determine the protein profile. The GSDMD-interacting proteins identified by mass spectrometry were used for gene ontology (GO) analysis, ggplot2 R package was used to draw bubble diagram of representative results of GO analysis. The network of proteins involved in ER-stress response was visualized by string database using default parameters, medium confidence (interaction score >0.400) was presented.

### Establishment of a chemotherapy model of transplanted tumours in nude mice

For the establishment of a nude mouse xenograft model, OSCC cells (1 × 10^7^ cells/150 µL) were implanted under the skin of the upper-right back of BALB/c nude mice (female, 5-weeks old). The tumour was measured every 3–4 days to determine the tumour volume. Mice were randomly assigned to different experimental groups.

After tumour formation, the experimental groups were designed as follows: the control group (Control), overexpression group (GSDMD), chemotherapy group (Cis) and PERK inhibitor–chemotherapy-combination group (PERK inhibitor–Cis). We regularly observed the growth of the nude mice and the size of the tumours and recorded the time of appearance. We began chemotherapy when the tumour was 0.5 cm^2^ in size (dose: 3 mg/kg, injected once every 4 days, 5 times in total). The tumour size (mm) was measured with Vernier callipers according to the formula: tumour volume (V)(mm3) = *π*/6 × long diameter (mm) × width diameter (mm)^2^. Tumour-growth curves were drawn according to the calculated tumour sizes. For the animal experiments, there were 6 nude mice per group, the injection cycle was once every 4 days for 5 cycles and the treatments were administered when the tumour size reached 200 mm^3^. The drug dosages were as follows: cisplatin: 3 mg/kg, once every 4 days, 5 times total; PERK inhibitor: 10 nM/kg, once every 4 days, 5 times total.

### Removal and detection of transplanted tumour

After the mice were divided into groups, they were sacrificed by cervical dislocation under ether anaesthesia; the subcutaneous tumours of the mice were used for pathological examination, and the size and weight of the tumours were measured. Immunohistochemistry (IHC), TUNEL staining, western blotting and other experimental techniques were applied to detect the expression of GSDMD–eIF2α-pathway proteins in the transplanted tumour tissue. Routine paraffin-embedded sections of tumours were subjected to immunohistochemical detection using the same method as above. After the tumour-body was ground, the tumour body homogenate was obtained to extract the protein, and western blotting experiments were performed using the method described above.

### Patient samples

Clinical patient-biopsy specimens were obtained according to hospital and ethical regulations. The sections were used for immunohistochemical staining; the staining and statistical methods were the same as before, and the positive staining rate was calculated. The sections were also used for TUNEL staining; the methods and statistical methods were the same as above, and the fluorescence rate was calculated. First, the tumour tissue was identified in the 40x field of view; at least three fields of view in the 100x field of view were assessed, and statistical analysis was performed. Patients who received neoadjuvant chemotherapy were divided into a response group and a nonresponse group according to the residual rate of tumour cells noted in the postoperative pathology report (those with a residual rate higher than 50% were included in the nonresponse group; otherwise, patients were included in the response group).

### IHC

Regarding antibodies, primary antibodies against proteins related to the GSDMD–eIF2α pathway (GSDMD, eIF2α, GAPDH, P-eIF2α, XBP-1, etc.) were applied for IHC analysis.

Regarding specimens, oral cancer and adjacent tissue specimens (fixed with 10% neutral formalin, routinely dehydrated and paraffin-embedded with a thickness of 4 μm) were assessed.

The IHC experimental process was as follows.

The paraffin sections were placed in an oven at 60 °C for 60 min, and then the routine dewaxing protocol was followed. Subsequently, the samples were washed with water and incubated with haematoxylin and eosin (HE). For antigen retrieval, 0.01 M sodium citrate buffer was added, and the samples were placed in a microwave oven to heat them. They were allowed to cool to room temperature naturally and then washed with PBS for 3 min in triplicate. H_2_O_2_ (3%) was added dropwise, incubated at 37 °C for 15 min and washed with PBS for 3 min in triplicate. Goat serum-blocking solution (10%) was added dropwise and incubated at 37 °C for 15 min. The solution was removed, and the primary antibody, diluted in an appropriate ratio (1:100 dilution), was dropped onto the sample. PBS was added dropwise to the blank control overnight at 4 °C and then washed with PBS for 5 min in triplicate. The fluorescent secondary antibody (antibody-dilution ratio was 1:100) was added dropwise, incubated at 37 °C in the dark for 60 min and washed in PBS for 5 min in triplicate. DAPI working solution was added dropwise, incubated at 37 °C for 10 min and washed 3 times with PBS. The cells were mounted with an anti-fluorescence quenching mount and allowed to stand for 5 min in the dark at room temperature. IHC detection was performed using a nonfluorescent secondary antibody and DAB counterstaining. All of the results were judged and counted by two or more independent pathologists.

### TUNEL fluorescent-label staining

The sections were routinely deparaffinized, and the reaction solution was configured according to the instructions of Beyotime’s TUNEL Kit (C1086). The sections were incubated in the dark at 37 °C for 60 min. After mounting the slide with an anti-fluorescence quencher, we observed the slides and obtained pictures. The 100x field of view and the 200x field of view were assessed separately, and the 200x field of view was used for statistical analysis.

### Statistical analyses

All of the statistical analyses were conducted using SPSS statistical software, version 19.0. Chi-square analyses were used to examine the correlation between GSDMD expression and tumour status in tumour and adjacent normal-control (ANC) tissues. Fisher’s exact test was used to assess the relationships between GSDMD expression and xenograft mouse sample features. Student’s *t*-test was used to compare the PCR, cell apoptosis, tumour xenograft and cell-function results (proliferation, migration, invasion, etc.) between the different groups. Unless otherwise noted, quantitative data are expressed as the mean and standard deviation (SD). Statistical significance was determined with Student’s paired *t*-test (*P < 0.05; **P < 0.01; ***P < 0.001, compared with the controls).

## Supplementary information

Supplementary material

## Data Availability

The datasets used for the current study are available from the corresponding author on reasonable request. All of the data generated or analysed during this study are included in this published article and its supplementary information files
