# Supplementary material for: GSDMD enhances cisplatin-induced apoptosis by promoting the phosphorylation of eIF2α and activating the ER-stress response
Source: Cell Death Discov. 2022 Mar 14;8:114. doi: 10.1038/s41420-022-00915-8 (PMC8921229; doi:10.1038/s41420-022-00915-8)

Figure S1. Changes in IC50 of cisplatin after GSDMD was knocked down by siRNA in cells with high GSDMD expression.

a. After using different siRNAs to knock down GSDMD in um1 cell lines, the IC50 of cisplatin was significantly increased.
b. B. After using different siRNAs to knock down GSDMD in Cal-33 cell lines, the IC50 of cisplatin was significantly increased.
c. C.After using different siRNAs to knock down GSDMD in the hela cell line, the IC50 of cisplatin was significantly increased.

Figure S2. GSDMD increases the cisplatin chemosensitivity of cells through a non-pyroptotic pathway.

a. After treatment with cisplatin for 24 h, the release of TNF-α in the medium was assessed by ELISA; the results showed that cisplatin treatment did not increase the release of IL-1β in Cal-27 and SCC-9 cells.

Figure S3. Statistical analysis results of the ratio of pyroptotic cells to total cells in live-image observation.

The statistical results showed that in each experimental group, no matter whether GSDMD was overexpressed or not, no pyroptosis occurred after cisplatin treatment. In the two positive controls, about 50% of the cells had undergone pyroptosis.

Figure S4. Western blotting results showed that the expression of NLRP-3 in the two head and neck squamous cell carcinoma cell lines (Cal-27 and HSC-6) was low or absent. Human monocytes (THP-1) and human oral mucosal epithelial cells (hok) were used as positive controls.

Figure S5. The expression levels of multiple NLRs and TLRs in tumor cells.

a. NLRP1 is highly expressed in SCC-9 and Cal-27 ($p<0.001$), compared with human monocytes.
b. B. TLR4 is highly expressed in SCC-9 ($p<0.001$). However, the expression is low in CAL-27 and Hela ($p<0.01$), compared with human monocytes.
c. C. NLRP1 is almost not expressed in SCC-9 and Cal-27 ($p<0.001$).
d. D. The expression of TLR9 in SCC-9 and Cal-27 is low ($p<0.001$).

Figure S6. KEGG analysis of mass spectrometry results

Figure S7. The phosphorylation of eIF2α in CAL-27 cells overexpressing GSDME.

Western blot showed that the phosphorylation level of eIF2α in cells

overexpressing GSDME did not change significantly.

Figure S8. Western blot was used to detect the phosphorylation levels of GSDMD and eIF2α.
Western blot results show that cisplatin can increase the level of eIF2α phosphorylation, and PERK inhibitor can eliminate this change.

Figure S9. PERK knockdown efficiency of siRNA and detection of apoptosis level after PERK knockdown.

a. Western blotting showed that of the two PERK siRNAs, si1# can significantly knock down the expression of PERK.
b. The results of flow cytometry detection of apoptosis showed that tumor cells after knocking down PERK, including CAL-27 and SCC-9, showed lower levels of apoptosis in cisplatin treatment.

Figure S10. The tumor homogenate protein after chemotherapy of transplanted tumor in nude mice was used in immunoblotting experiments.
The results showed that the use of PERK inhibitors can reduce the phosphorylation level of eIF2α.

Figure S11. Effect of GSDMD on eIF2α phosphorylation in non-cisplatin chemotherapy
The phosphorylation level of eIF2α at 0, 4, 6, and 12 hours after the use of TG in the control group and overexpression group. The phosphorylation level of eIF2α was higher in the overexpression group at 0h. The higher phosphorylation level of eIF2α at 4h peaked in both groups. At 6h, the phosphorylation level of eIF2α in the overexpression group decreased more rapidly.

Figure S1

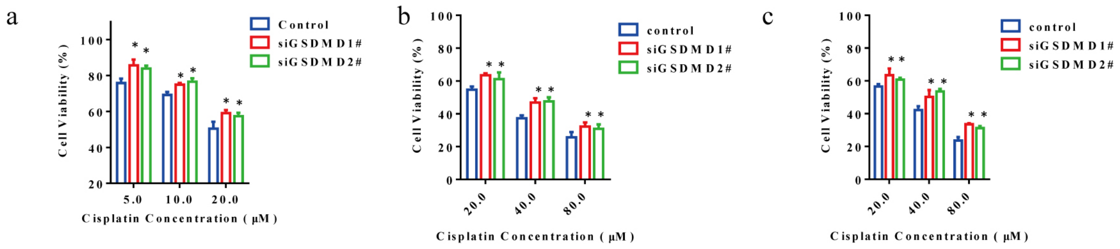
a
Cell Viability (%)
Control
siGSDMD1#
siGSDMD2#
5.0
10.0
20.0
Cisplatin Concentration (μM)
b
Cell Viability (%)
control
siGSDMD1#
siGSDMD2#
20.0
40.0
80.0
Cisplatin Concentration (μM)
c
Cell Viability (%)
control
siGSDMD1#
siGSDMD2#
20.0
40.0
80.0
Cisplatin Concentration (μM)

Figure S2

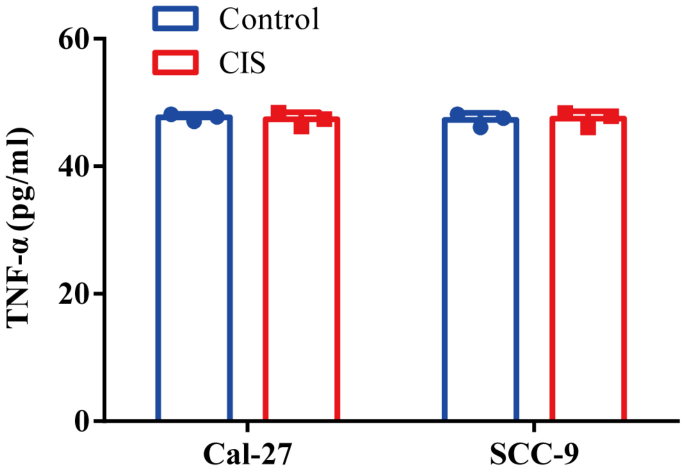

Figure S3

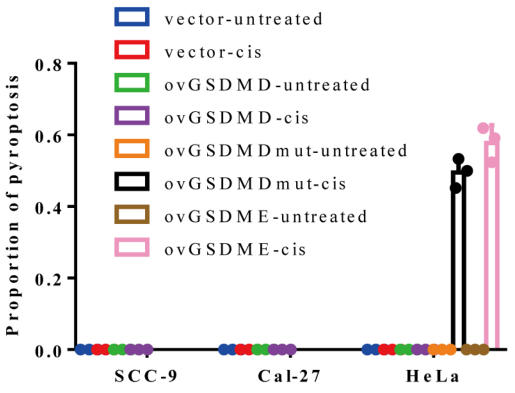
vector-untreated
vector-cis
ovGSDMD-untreated
ovGSDMD-cis
ovGSDMDmut-untreated
ovGSDMDmut-cis
ovGSDME-untreated
ovGSDME-cis
Proportion of pyroptosis
0.8
0.6
0.4
0.2
0.0
SCC-9
Cal-27
HeLa

Figure S4

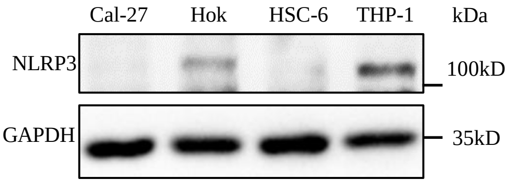

Figure S5

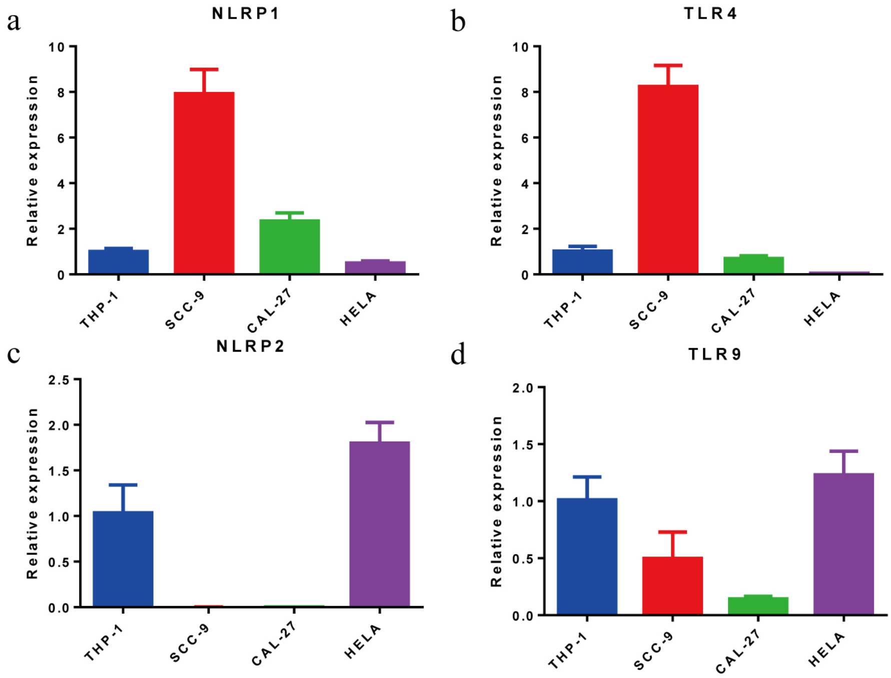
a
NLRP1
Relative expression
10
8
6
4
2
0
THP-1
SCC-9
CAL-27
HELA
b
TLR4
Relative expression
10
8
6
4
2
0
THP-1
SCC-9
CAL-27
HELA
c
NLRP2
Relative expression
2.5
2.0
1.5
1.0
0.5
0.0
THP-1
SCC-9
CAL-27
HELA
d
TLR9
Relative expression
2.0
1.5
1.0
0.5
0.0
THP-1
SCC-9
CAL-27
HELA

Figure S6

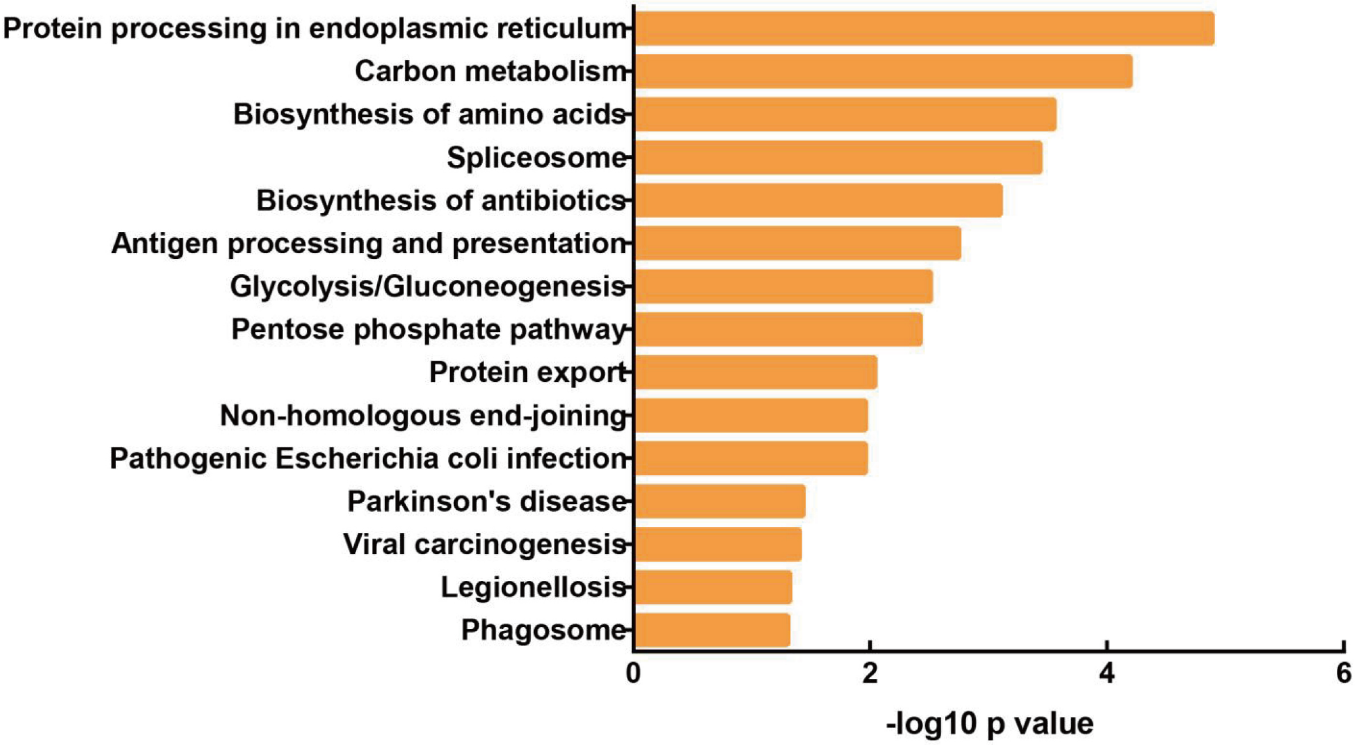
Protein processing in endoplasmic reticulum
Carbon metabolism
Biosynthesis of amino acids
Spliceosome
Biosynthesis of antibiotics
Antigen processing and presentation
Glycolysis/Gluconeogenesis
Pentose phosphate pathway
Protein export
Non-homologous end-joining
Pathogenic Escherichia coli infection
Parkinson's disease
Viral carcinogenesis
Legionellosis
Phagosome
0
2
4
6
-log10 p value

Figure S7

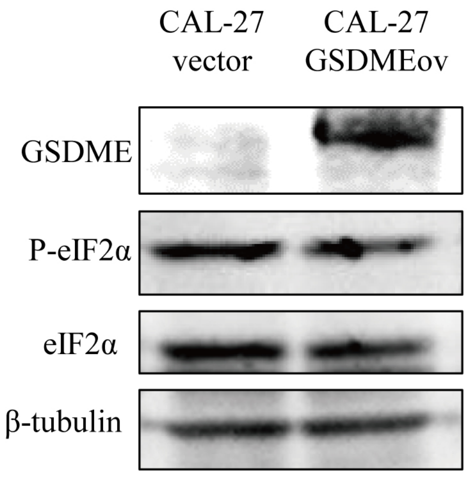

Figure S8

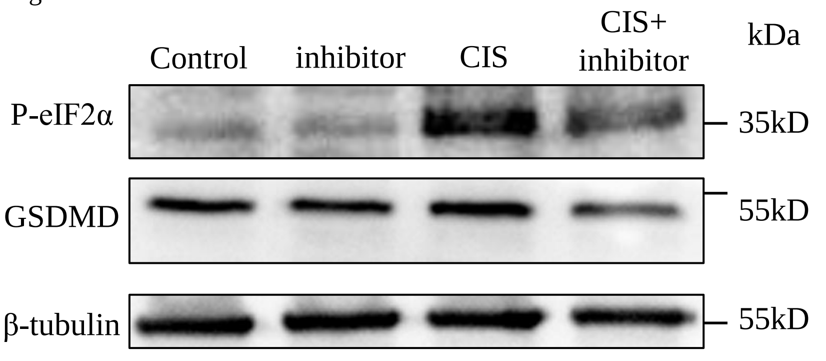
Control
inhibitor
CIS
CIS+ inhibitor
kDa
P-eIF2α
35kD
GSDMD
55kD
β-tubulin
55kD

Figure S9

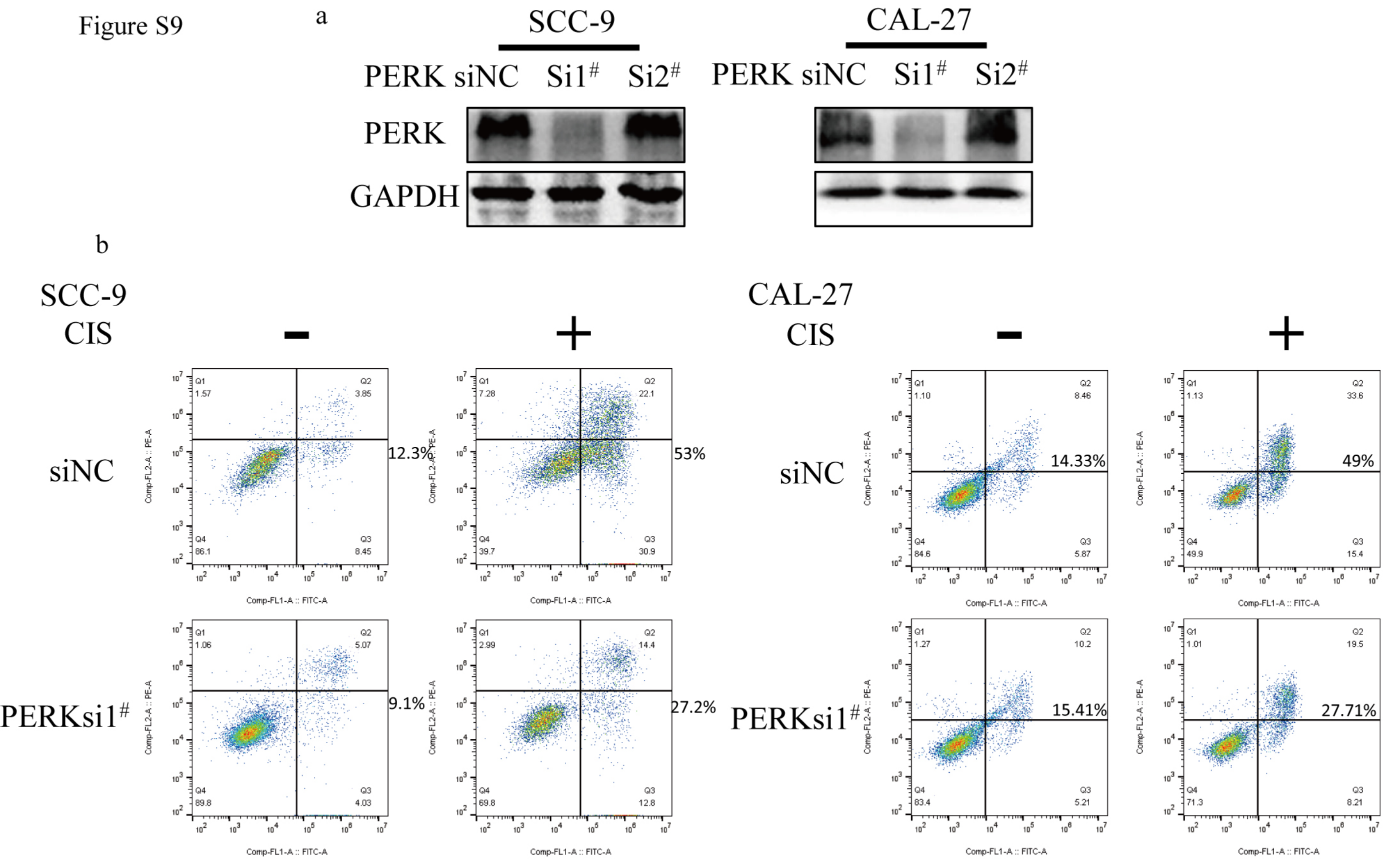

a
SCC-9
CAL-27
PERK siNC
Si1#
Si2#
PERK siNC
Si1#
Si2#
PERK
GAPDH
b
SCC-9
CIS
CAL-27
CIS
siNC
PERKsi1#
siNC
PERKsi1#
12.3%
53%
9.1%
27.2%
14.33%
49%
15.41%
27.71%
Comp-FL1-A :: FITC-A
Comp-FL2-A :: PE-A

Figure S10

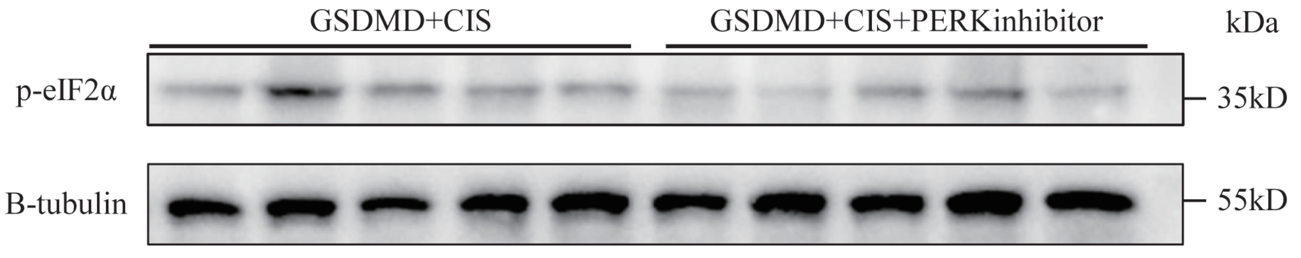
GSDMD+CIS
GSDMD+CIS+PERKinhibitor
kDa
p-eIF2α
35kD
B-tubulin
55kD

Figure S11

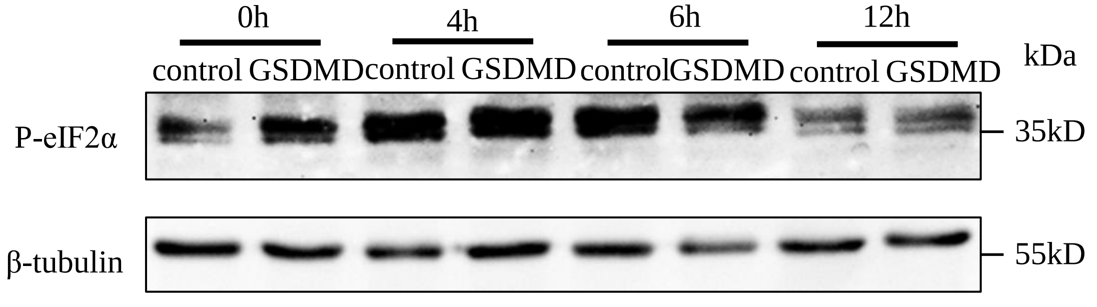
0h
4h
6h
12h
kDa
control
GSDMD
control
GSDMD
control
GSDMD
control
GSDMD
P-eIF2α
35kD
β-tubulin
55kD

IGE BIOTECH INC, LTD.

CELL LINE AUTHENTICATION SERVICE

STR Profile Report

**Sample Submitted By:**	**HUANG ZI XIAN**
**Email Address:**	**258001917@qq.com**
**Project Number:**	**IGXN21174**
**Cell Line Designation:**	**SCC-9**
**Date Sample Received:**	**2021-08-15**
**Date Reported:**	**2021-08-18**
**Methodology：**	The cell line sample was processed and 21 short tandem repeat (STR) loci including the gender determining locus, Amelogenin, were amplified using the IGE STE 21 commercial kit in an ABI 9700 PCR system. The PCR product was processed using an ABI Prism® 3730XL Genetic Analyzer. Data were analyzed using GeneMapper® ID-X v1.5 software (Applied Biosystems). Appropriate positive and negative controls were run and confirmed for each sample submitted.
**Data Interpretation:**	Cell lines were authenticated using Short Tandem Repeat (STR) analysis as described in 2012 in ANSI Standard (ASN-0002) by the ATCC Standards Development Organization (SDO) and in Capes-Davis et al., Match criteria for human cell line authentication: Where do we draw the line? Int J Cancer. 2013;132(11):2510-9.

Results

Alleles / STR profile	Allele 1	Allele 2	Allele 3	Allele 4
Amelogenin	X	Y		
D3S1358	15	15		
vWA	17	17		
D7S820	8	8		
CSF1PO	11	11		
PentaE	11	11		
D8S1179	13	13		
D21S11	28	28		
D16S539	10	11		
D2S1338	19	21		
PentaD	9	9		
D19S433	12	14		

IGE BIOTECH INC, LTD.

CELL LINE AUTHENTICATION SERVICE

STR Profile Report

Alleles / STR profile	Allele 1	Allele 2	Allele 3	Allele 4
TH01	8	9		
D13S317	9	9		
TPOX	9	11		
D18S51	12	14		
D6S1043	11	18		
D1S1656	15	17		
D5S818	12	12		
D12S391	20	20		
FGA	20	25		

Cell lines with ≥80% match are considered to be related; i.e., derived from a common ancestry. Cell lines with between a 55% to 80% match require further profiling for authentication of relatedness.

☐The submitted sample profile is human, but not a match for any profile in the DSMZ/ATCC/STR STR databases which include approximately 2455 different type of cells.

☑The submitted profile is an exact match for the following IGE human cell line(s) in the IGE STR database (8 core loci plus Amelogenin): SCC-9

☐ The submitted profile is similar to the following human cell line(s):

Matches:

NO.	Percent Match	Cell No.	Cell name	D5S818	D13S317	D7S820	D16S539	vWA	TH01	AM	TPOX	CSF1PO
			Query(Your Cell)	12,12	9,9	8,8	10,11	17,17	8,9	X,Y	9,11	11,11
1	100%		SCC-9	12,12	9,9	8,8	10,11	17,17	8,9	X,Y	9,11	11,11
2	100%	CRL-1629	SCC-9	12,12	9,9	8,8	10,11	17,17	8,9	X,Y	9,11	11,11

IGE BIOTECH INC, LTD.

CELL LINE AUTHENTICATION SERVICE

STR Profile Report

STR Typing

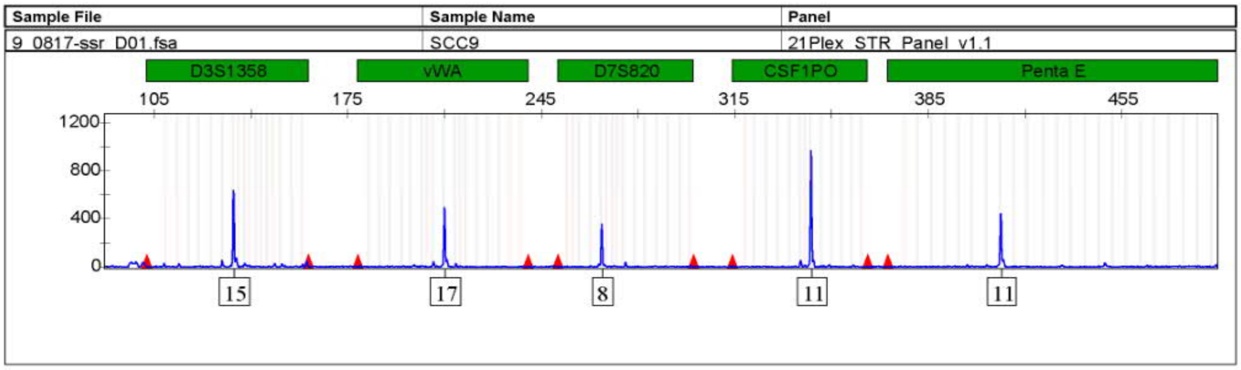

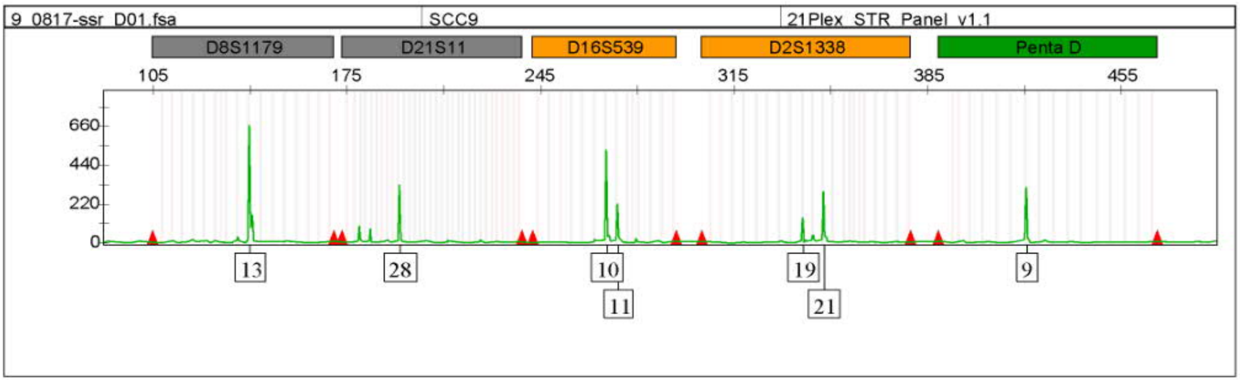

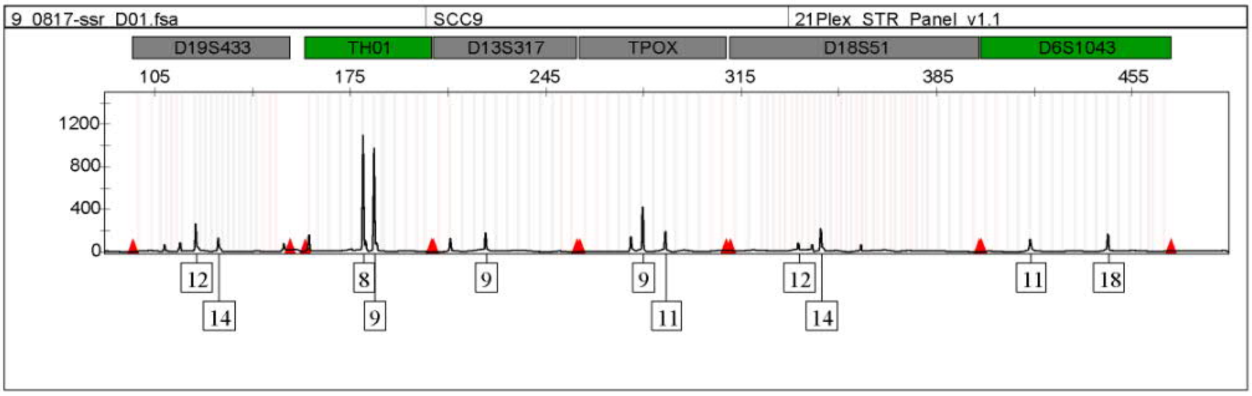

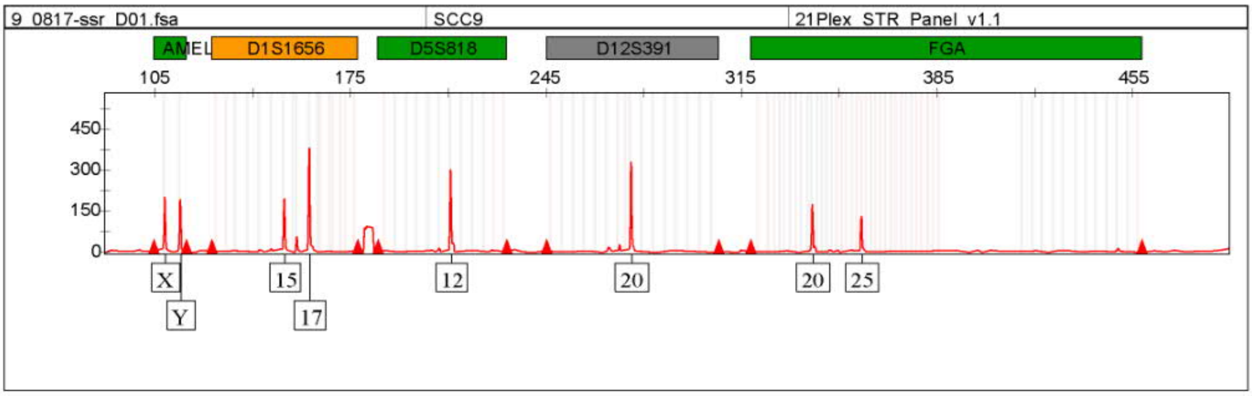

Disclaimer:

IGE Biotechnology performs STR Profiling following ISO 9001:2008 and ISO/IEC 17025:2005 quality standards. There are no warranties with respect to the services or results supplied, express or implied, including, without limitation, any implied warranty of merchantability or fitness for a particular purpose. IGE Biotechnology is not liable for any damages or injuries resulting from receipt and/or improper, inappropriate, negligent or other wrongful use of the test results supplied, and/or from misidentification, misrepresentation, or lack of accuracy of those results. Your exclusive remedy against IGE Biotechnology and those supplying materials used in the services for any losses or damage of any kind whatsoever, whether in contract, tort, or otherwise, shall be, at IGE Biotechnology's option, refund of the fee paid for such service or repeat of the service.

Contact:

IGE Biotechnology Inc. LTD.
020-89053723
info@igebio.com
12 3rd Luoxuan Rd., Building 4 Room 505
Guangzhou International Biotech Island
Guangzhou, China 510320
http://www.igebio.com

IGE BIOTECH INC, LTD.

CELL LINE AUTHENTICATION SERVICE

STR Profile Report

**Sample Submitted By:**	**HUANG ZI XIAN**
**Email Address:**	**258001917@qq.com**
**Project Number:**	**IGXN21174**
**Cell Line Designation:**	**SCC-9**
**Date Sample Received:**	**2021-08-15**
**Date Reported:**	**2021-08-18**
**Methodology：**	The cell line sample was processed and 21 short tandem repeat (STR) loci including the gender determining locus, Amelogenin, were amplified using the IGE STE 21 commercial kit in an ABI 9700 PCR system. The PCR product was processed using an ABI Prism® 3730XL Genetic Analyzer. Data were analyzed using GeneMapper® ID-X v1.5 software (Applied Biosystems). Appropriate positive and negative controls were run and confirmed for each sample submitted.
**Data Interpretation:**	Cell lines were authenticated using Short Tandem Repeat (STR) analysis as described in 2012 in ANSI Standard (ASN-0002) by the ATCC Standards Development Organization (SDO) and in Capes-Davis et al., Match criteria for human cell line authentication: Where do we draw the line? Int J Cancer. 2013;132(11):2510-9.

Results

Alleles / STR profile	Allele 1	Allele 2	Allele 3	Allele 4
Amelogenin	X	Y		
D3S1358	15	15		
vWA	17	17		
D7S820	8	8		
CSF1PO	11	11		
PentaE	11	11		
D8S1179	13	13		
D21S11	28	28		
D16S539	10	11		
D2S1338	19	21		
PentaD	9	9		
D19S433	12	14		

CELL LINE AUTHENTICATION SERVICE

STR Profile Report

STR profile \ Alleles	Allele 1	Allele 2	Allele 3	Allele 4
TH01	8	9		
D13S317	9	9		
TPOX	9	11		
D18S51	12	14		
D6S1043	11	18		
D1S1656	15	17		
D5S818	12	12		
D12S391	20	20		
FGA	20	25		

Cell lines with ≥80% match are considered to be related; i.e., derived from a common ancestry. Cell lines with between a 55% to 80% match require further profiling for authentication of relatedness.

☐The submitted sample profile is human, but not a match for any profile in the DSMZ/ATCC/STR STR databases which include approximately 2455 different type of cells.

☑The submitted profile is an exact match for the following IGE human cell line(s) in the IGE STR database (8 core loci plus Amelogenin): SCC-9

☐ The submitted profile is similar to the following human cell line(s):

Matches:

NO.	Percent Match	Cell No.	Cell name	D5S818	D13S317	D7S820	D16S539	vWA	TH01	AM	TPOX	CSF1PO
			Query(Your Cell)	12,12	9,9	8,8	10,11	17,17	8,9	X,Y	9,11	11,11
1	100%		SCC-9	12,12	9,9	8,8	10,11	17,17	8,9	X,Y	9,11	11,11
2	100%	CRL-1629	SCC-9	12,12	9,9	8,8	10,11	17,17	8,9	X,Y	9,11	11,11

IGE BIOTECH INC, LTD.

CELL LINE AUTHENTICATION SERVICE

STR Profile Report

STR Typing

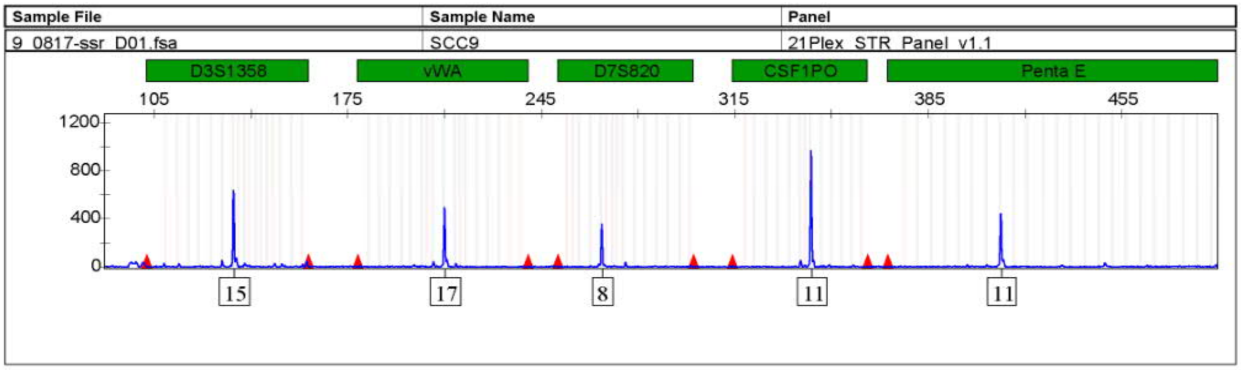

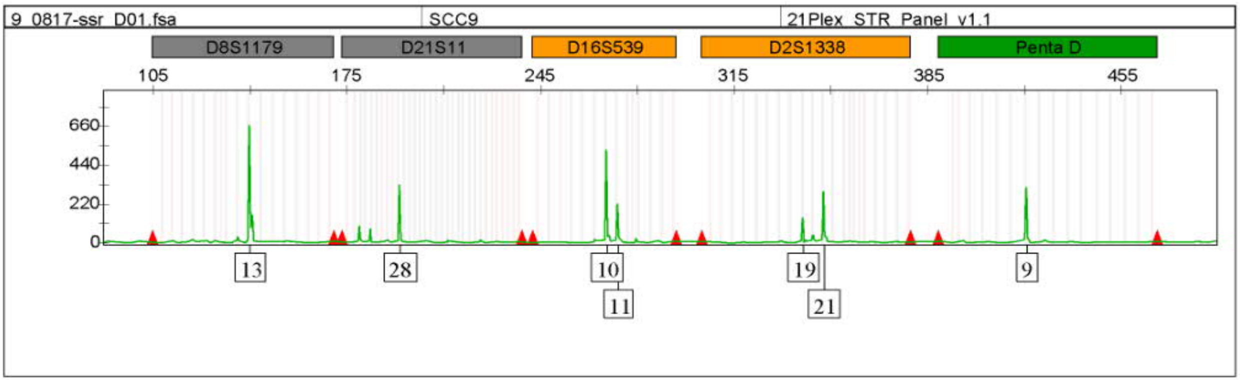

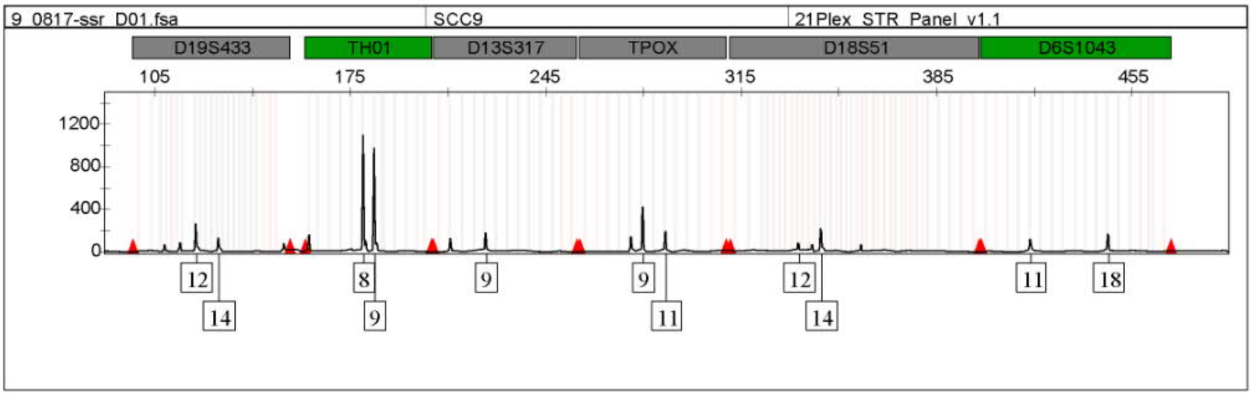

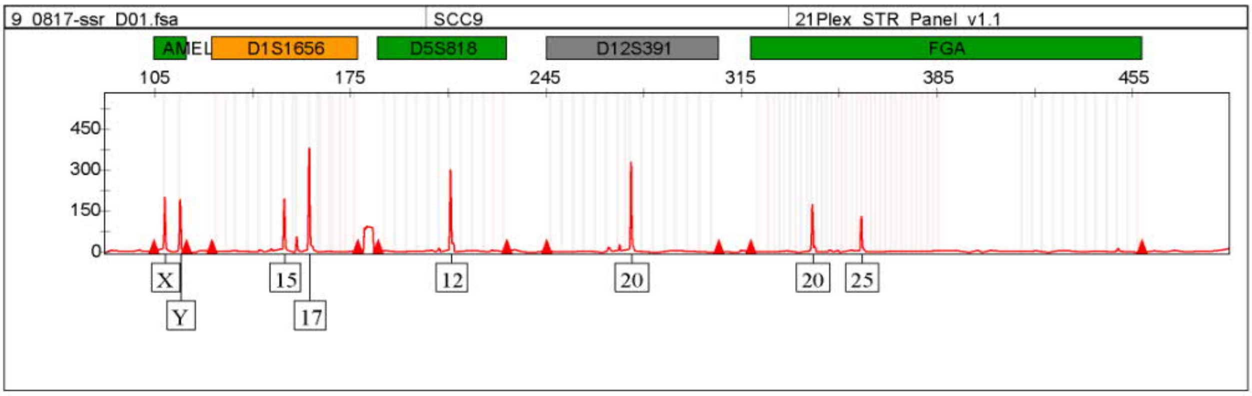

Disclaimer:

IGE Biotechnology performs STR Profiling following ISO 9001:2008 and ISO/IEC 17025:2005 quality standards. There are no warranties with respect to the services or results supplied, express or implied, including, without limitation, any implied warranty of merchantability or fitness for a particular purpose. IGE Biotechnology is not liable for any damages or injuries resulting from receipt and/or improper, inappropriate, negligent or other wrongful use of the test results supplied, and/or from misidentification, misrepresentation, or lack of accuracy of those results. Your exclusive remedy against IGE Biotechnology and those supplying materials used in the services for any losses or damage of any kind whatsoever, whether in contract, tort, or otherwise, shall be, at IGE Biotechnology's option, refund of the fee paid for such service or repeat of the service.

Contact:

IGE Biotechnology Inc. LTD.
020-89053723
info@igebio.com
12 3rd Luoxuan Rd., Building 4 Room 505
Guangzhou International Biotech Island
Guangzhou, China 510320
http://www.igebio.com

IGE BIOTECH INC, LTD.

CELL LINE AUTHENTICATION SERVICE

STR Profile Report

**Sample Submitted By:**	**HUANG ZI XIAN**
**Email Address:**	**258001917@qq.com**
**Project Number:**	**IGXN20028**
**Cell Line Designation:**	**CAL-27**
**Date Sample Received:**	**2020-04-01**
**Date Reported:**	**2020-04-03**
**Methodology：**	The cell line sample was processed and 21 short tandem repeat (STR) loci including the gender determining locus, Amelogenin, were amplified using the IGE STE 21 commercial kit in an ABI 9700 PCR system. The PCR product was processed using an ABI Prism® 3730XL Genetic Analyzer. Data were analyzed using GeneMapper® ID-X v1.5 software (Applied Biosystems). Appropriate positive and negative controls were run and confirmed for each sample submitted.
**Data Interpretation:**	Cell lines were authenticated using Short Tandem Repeat (STR) analysis as described in 2012 in ANSI Standard (ASN-0002) by the ATCC Standards Development Organization (SDO) and in Capes-Davis et al., Match criteria for human cell line authentication: Where do we draw the line? Int J Cancer. 2013;132(11):2510-9.

Results

STR profile \ Alleles	Allele 1	Allele 2	Allele 3	Allele 4
Amelogenin	X	X		
D3S1358	16	16		
vWA	14	17		
D7S820	10	10		
CSF1PO	10	12		
PentaE	7	7		
D8S1179	13	15		
D21S11	28	29		
D16S539	11	12		
D2S1338	23	24		
PentaD	9	10		
D19S433	14	15.2		

IGE BIOTECH INC, LTD.

CELL LINE AUTHENTICATION SERVICE

STR Profile Report

Alleles / STR profile	Allele 1	Allele 2	Allele 3	Allele 4
TH01	6	9.3		
D13S317	10	11		
TPOX	8	8		
D18S51	13	13		
D6S1043	12	12		
D1S1656	13	16		
D5S818	11	12		
D12S391	18.3	20		
FGA	25	25		

Cell lines with ≥80% match are considered to be related; i.e., derived from a common ancestry. Cell lines with between a 55% to 80% match require further profiling for authentication of relatedness.

☐The submitted sample profile is human, but not a match for any profile in the DSMZ/ATCC/STR STR databases which include approximately 2455 different type of cells.

☑The submitted profile is an exact match for the following IGE human cell line(s) in the IGE STR database (8 core loci plus Amelogenin): CAL-27

☐ The submitted profile is similar to the following human cell line(s):

Matches:

NO.	Percent Match	Cell No.	Cell name	D5S818	D13S317	D7S820	D16S539	vWA	TH01	AM	TPOX	CSF1PO
			Query(Your Cell)	11,12	10,11	10,10	11,12	14,17	6,9.3	X,X	8,8	10,12
1	100%	446	CAL-27	11,12	10,11	10,10	11,12	14,17	6,9.3	X,X	8,8	10,12
2	100%	446	CAL-27	11,12	10,11	10,10	11,12	14,17	6,9.3	X,X	8,8	10,12

IGE BIOTECH INC, LTD.

CELL LINE AUTHENTICATION SERVICE

STR Profile Report

STR Typing

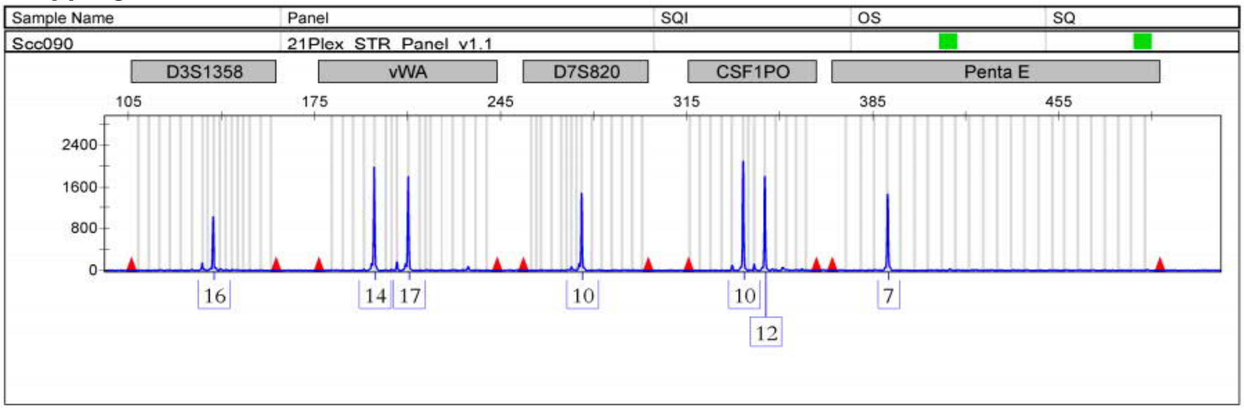

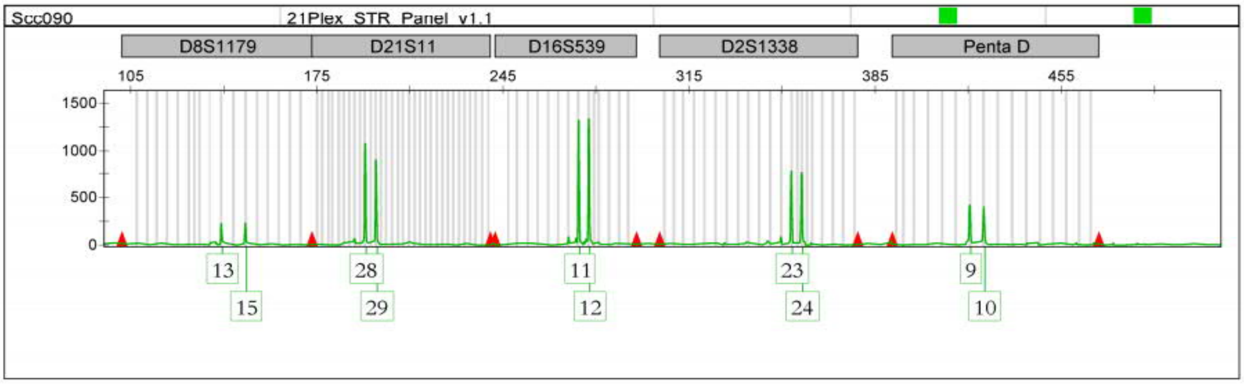

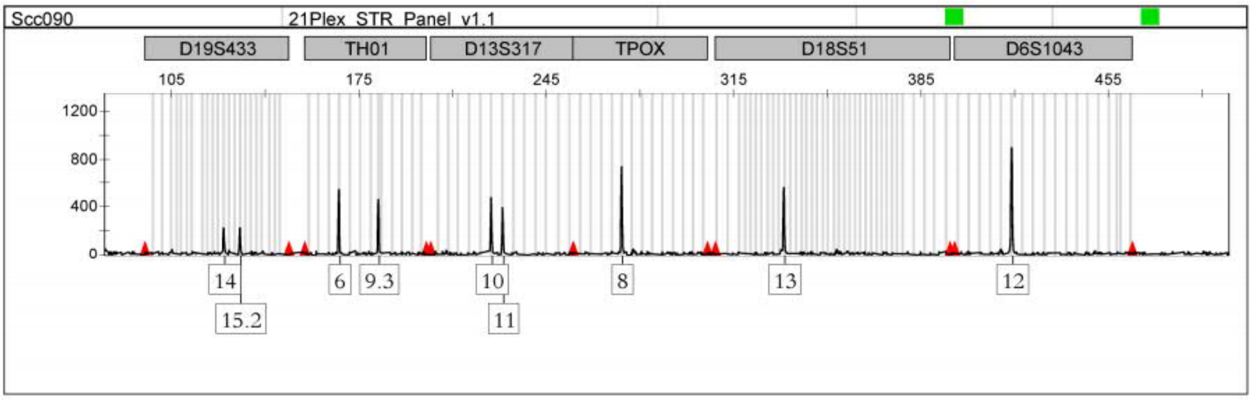

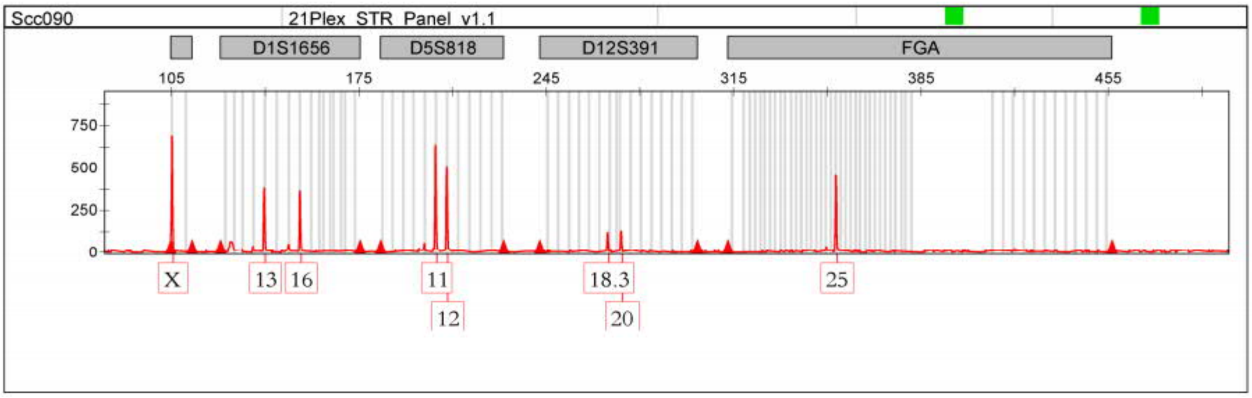

Disclaimer:

IGE Biotechnology performs STR Profiling following ISO 9001:2008 and ISO/IEC 17025:2005 quality standards. There are no warranties with respect to the services or results supplied, express or implied, including, without limitation, any implied warranty of merchantability or fitness for a particular purpose. IGE Biotechnology is not liable for any damages or injuries resulting from receipt and/or improper, inappropriate, negligent or other wrongful use of the test results supplied, and/or from misidentification, misrepresentation, or lack of accuracy of those results. Your exclusive remedy against IGE Biotechnology and those supplying materials used in the services for any losses or damage of any kind whatsoever, whether in contract, tort, or otherwise, shall be, at IGE Biotechnology's option, refund of the fee paid for such service or repeat of the service.

Contact:

IGE Biotechnology Inc. LTD.
020-89053723
info@igebio.com
12 3rd Luoxuan Rd., Building 4 Room 505
Guangzhou International Biotech Island
Guangzhou, China 510320
http://www.igebio.com

Original western blots

Figure 1F

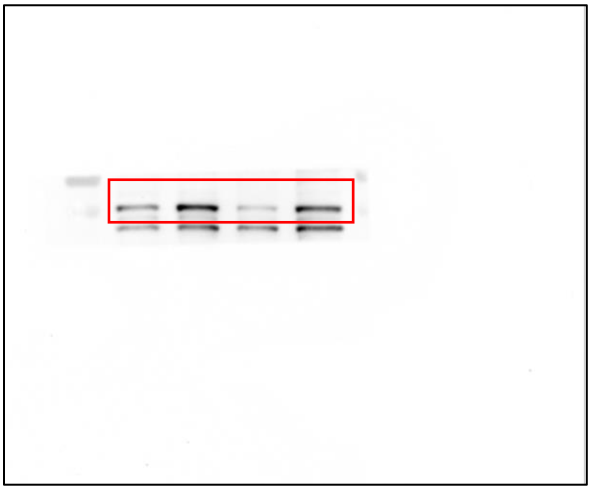

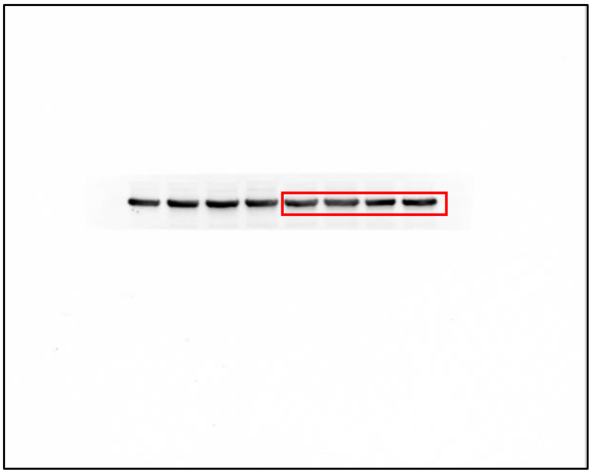

Figure 1I

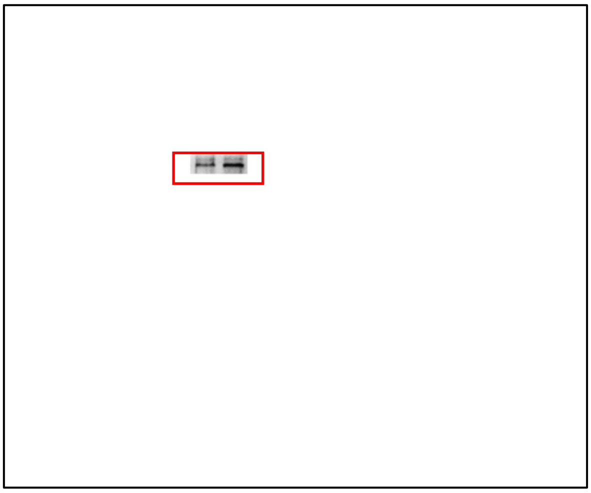

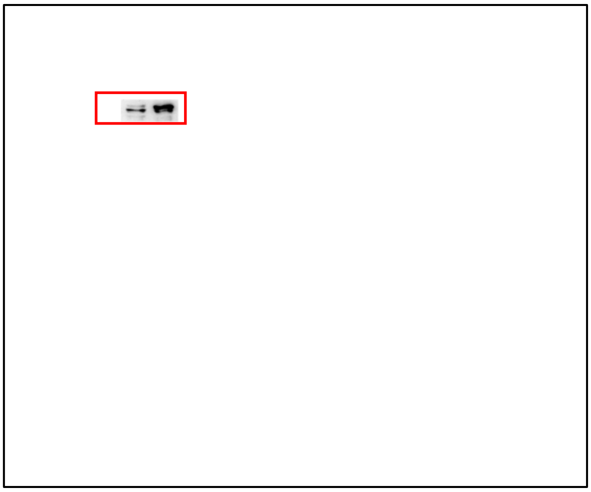

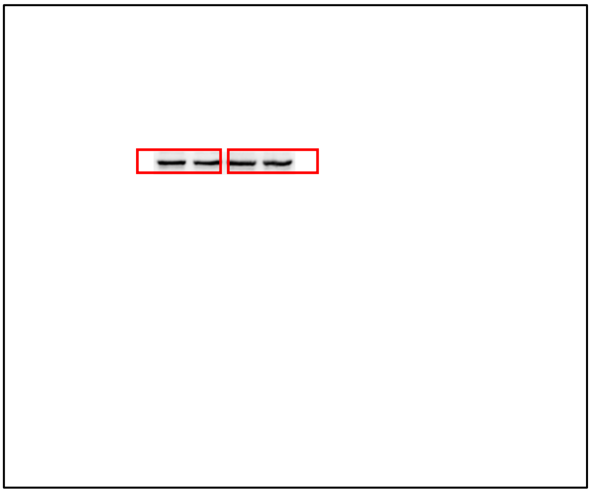

Figure 2C

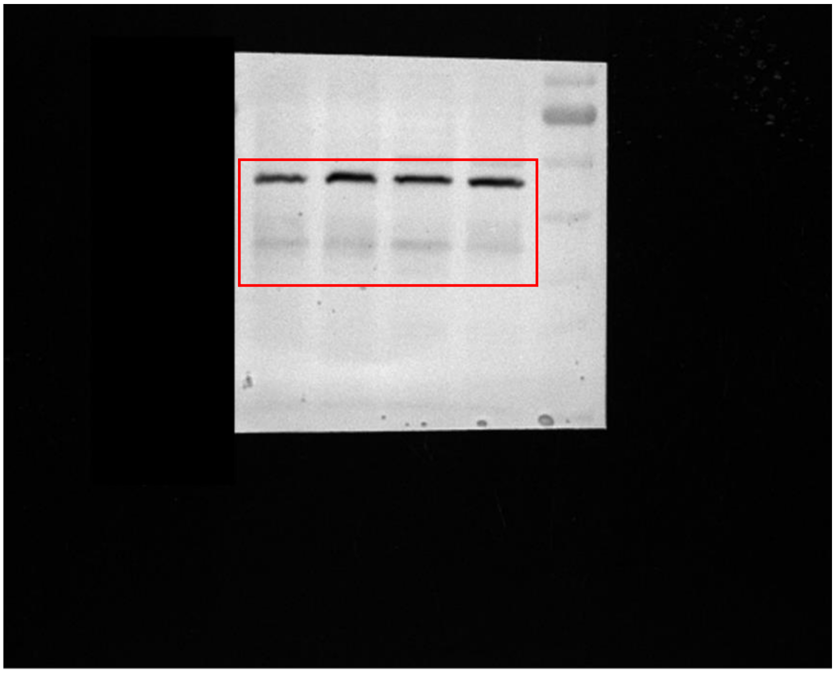

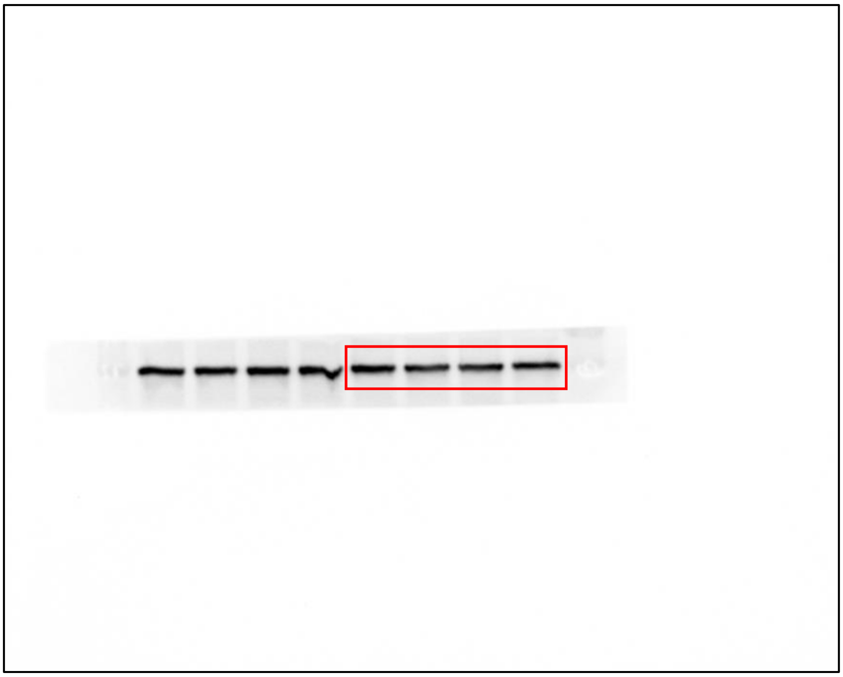

Figure 2G

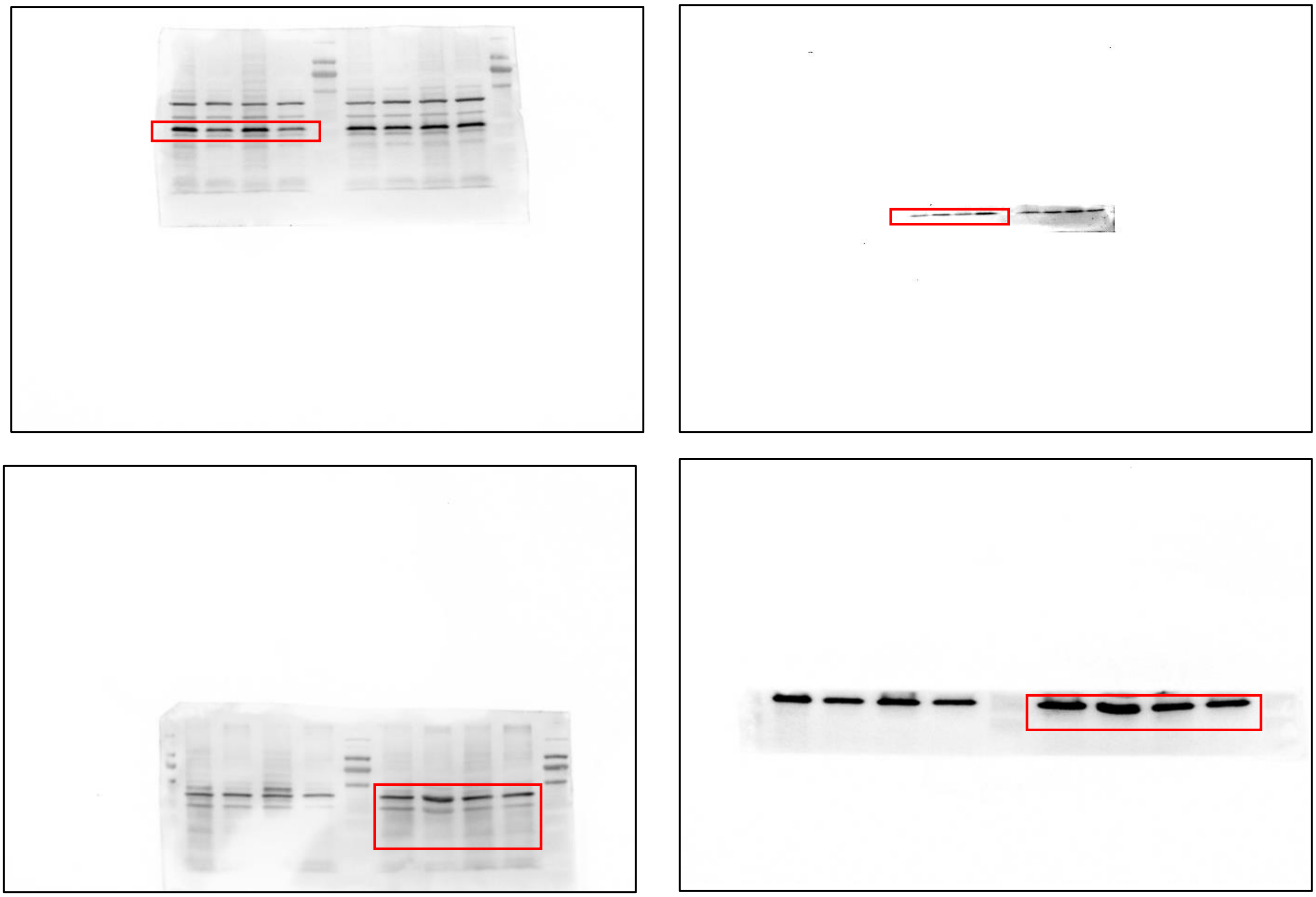

Figure 3A

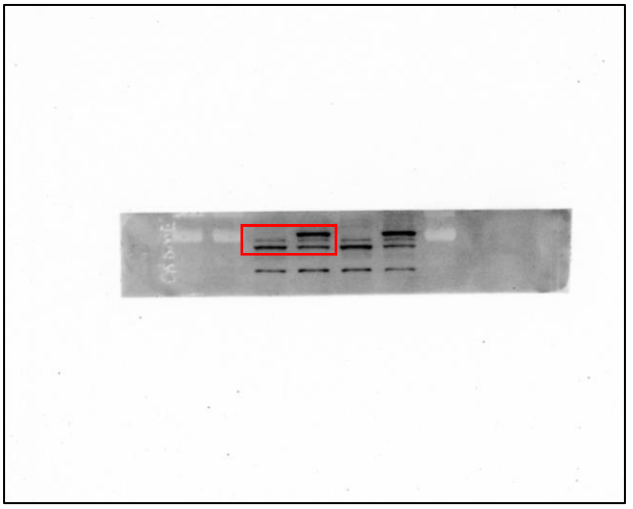

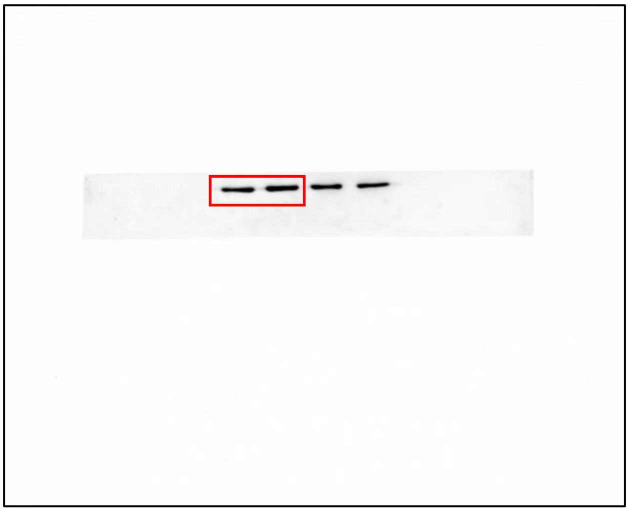

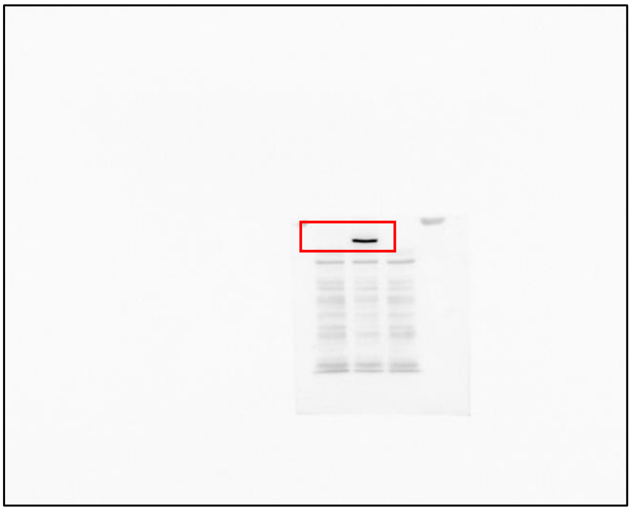

Figure 3D

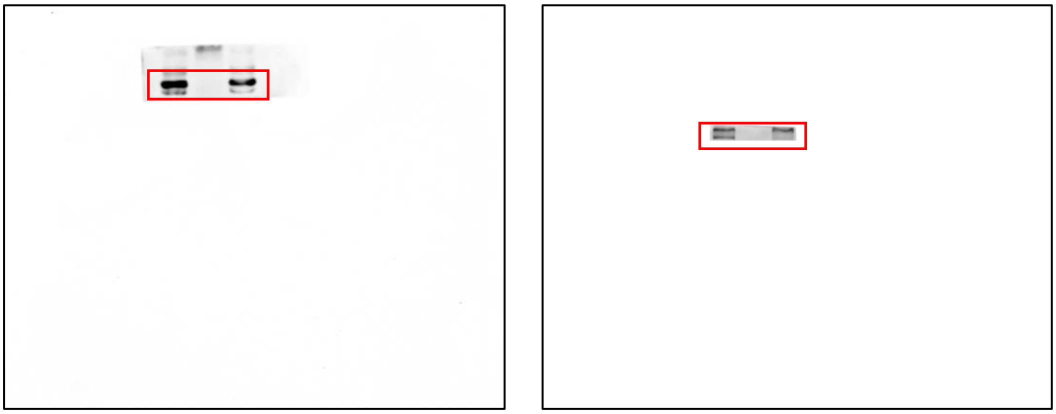

Figure 3E

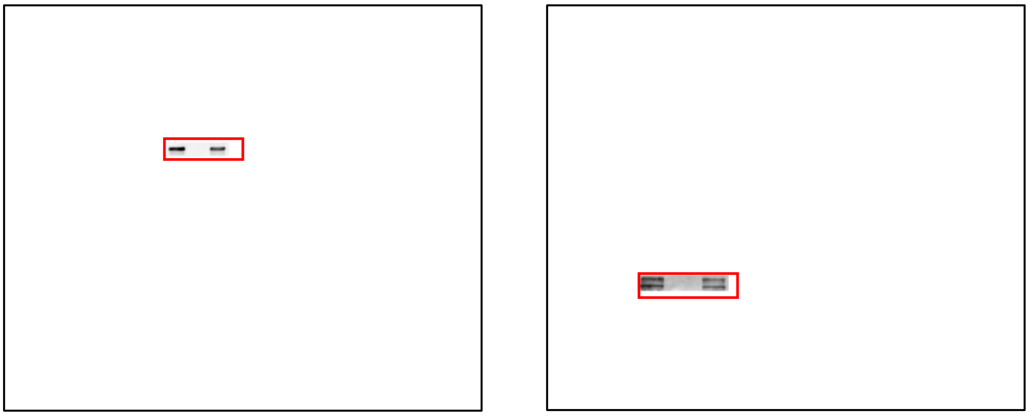

Figure 3F

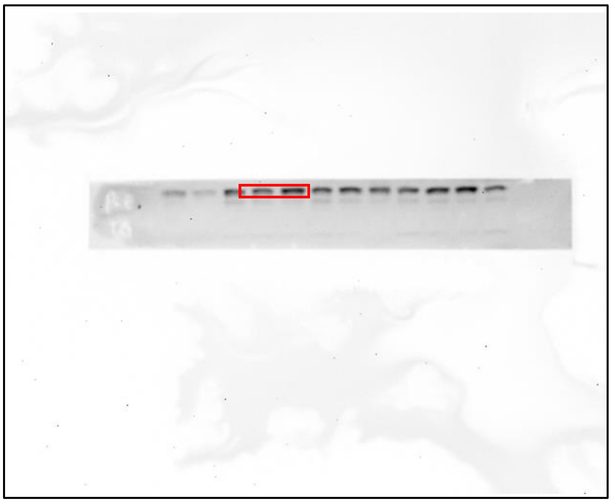

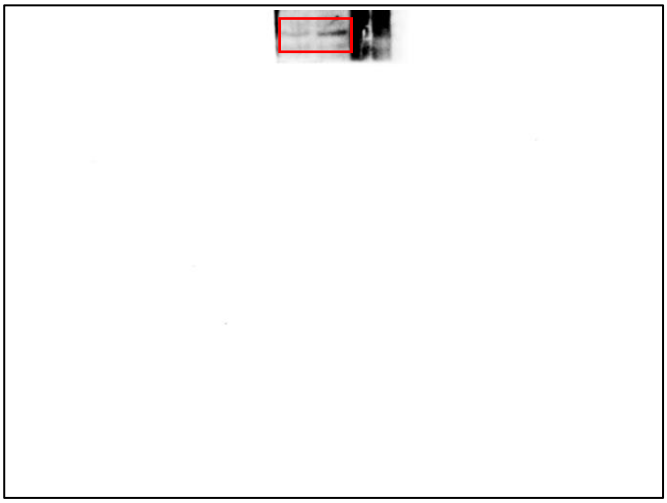

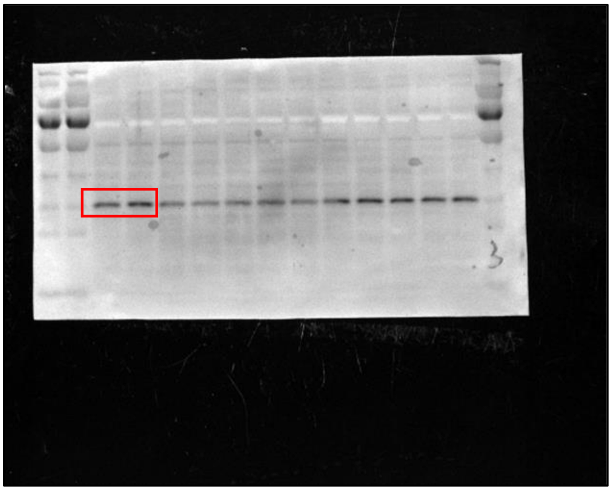

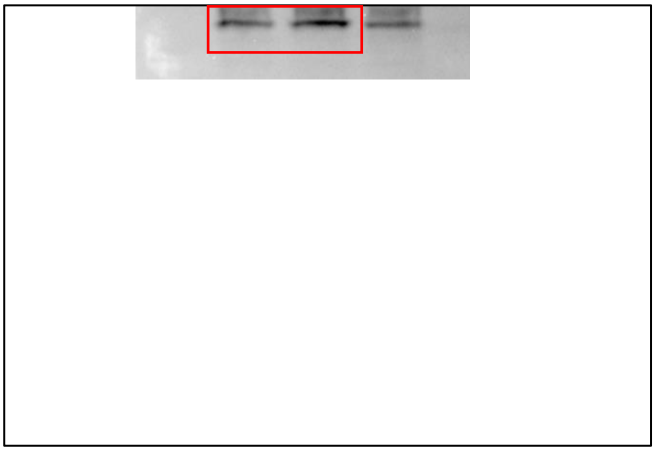

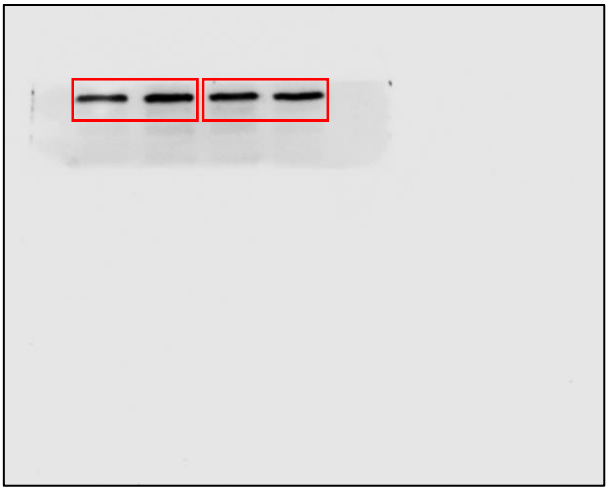

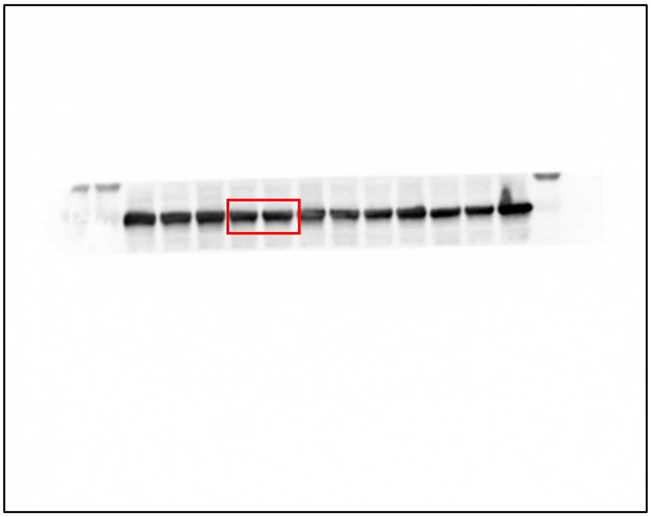

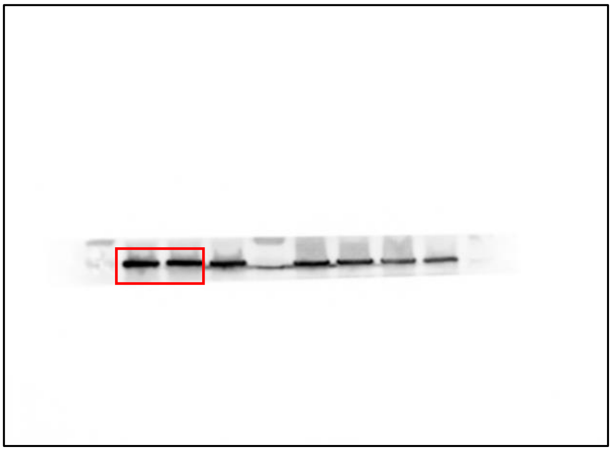

Figure 3G

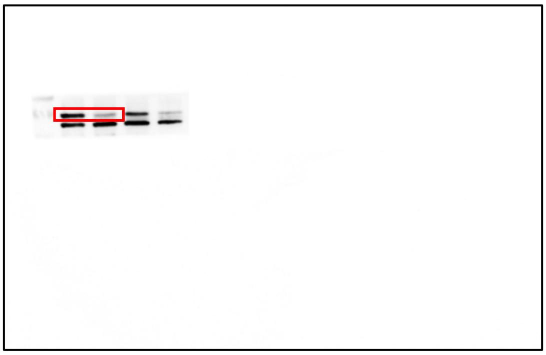

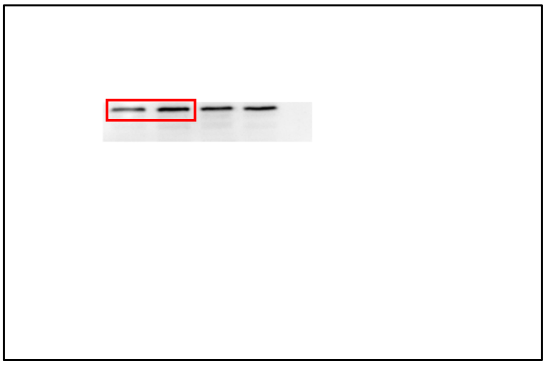

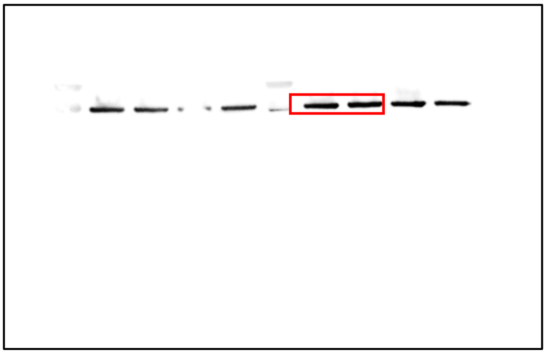

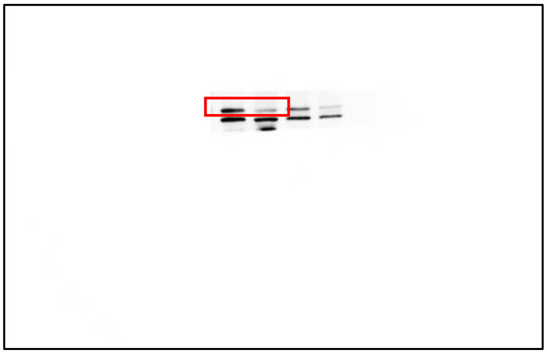

Figure 4A、B

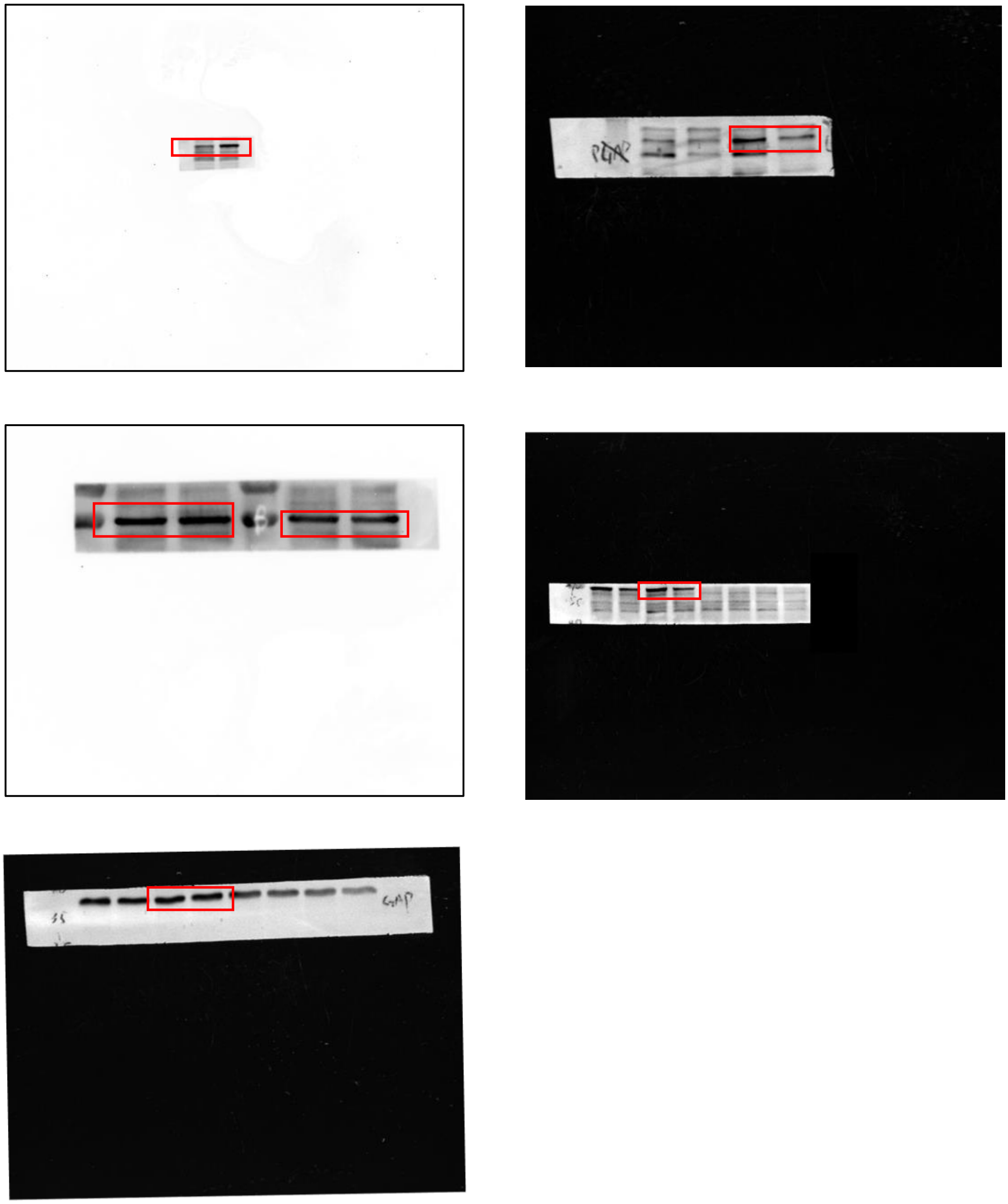

Figure 4C

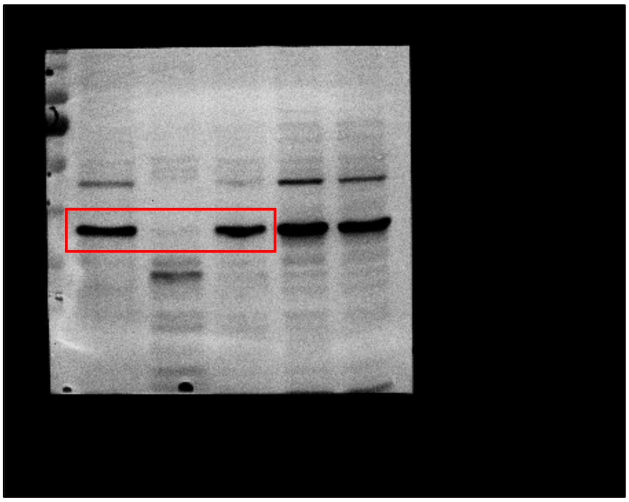

Figure 4D

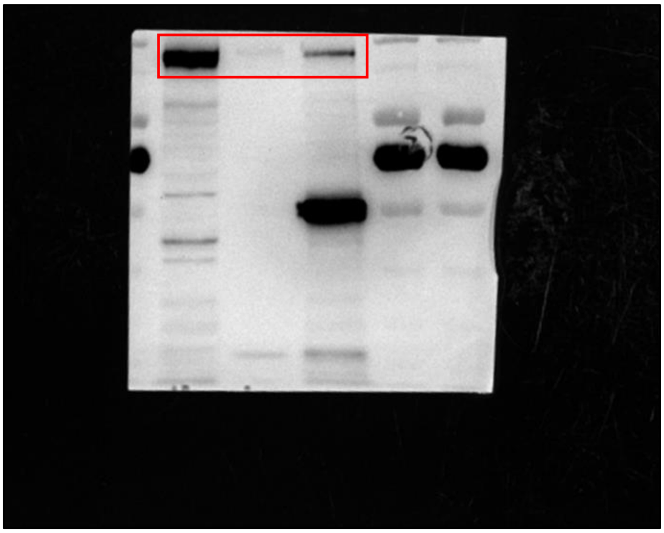

Figure 4E

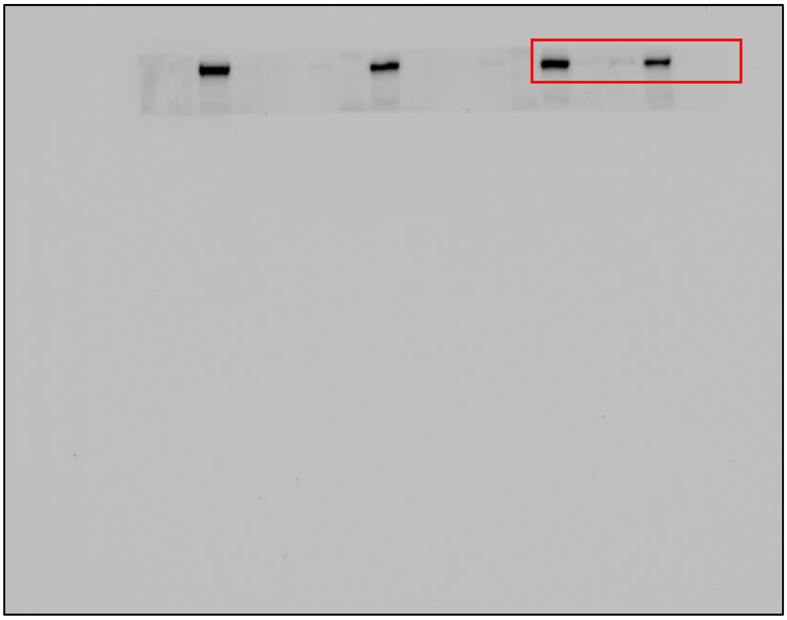

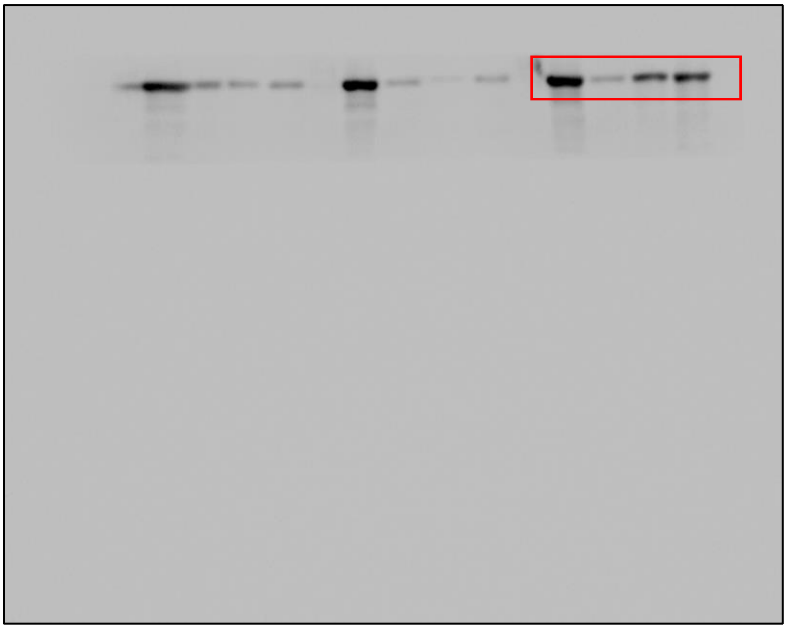

Figure 4F

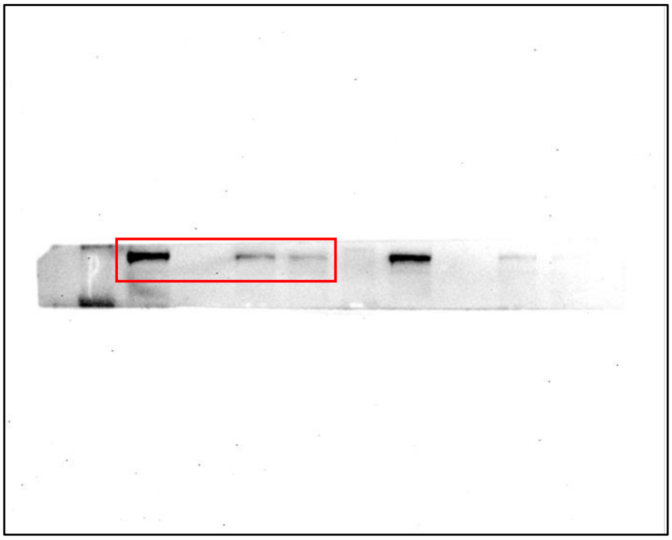

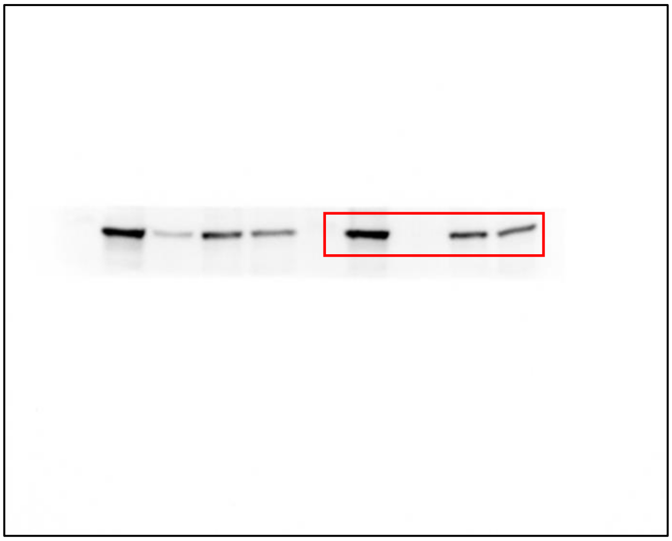

Figure 5A

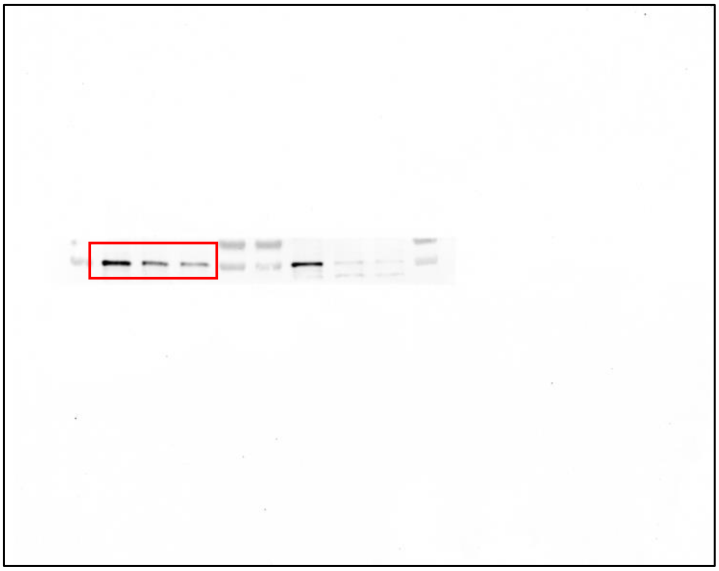

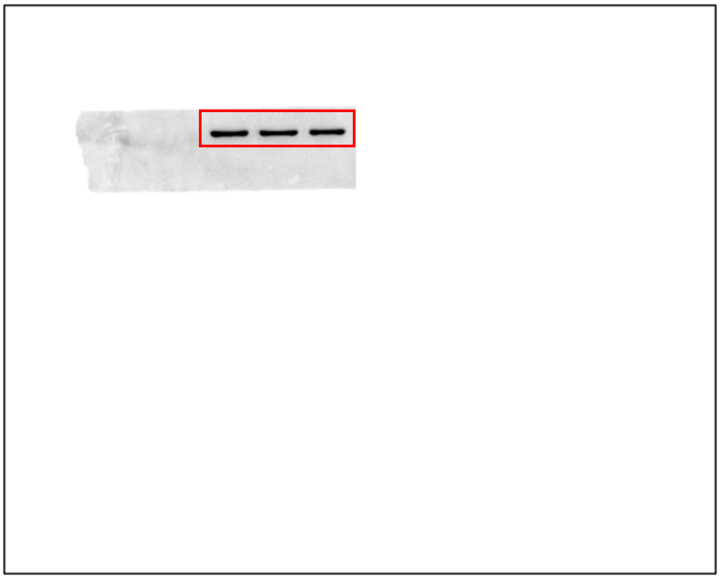

Figure 5B

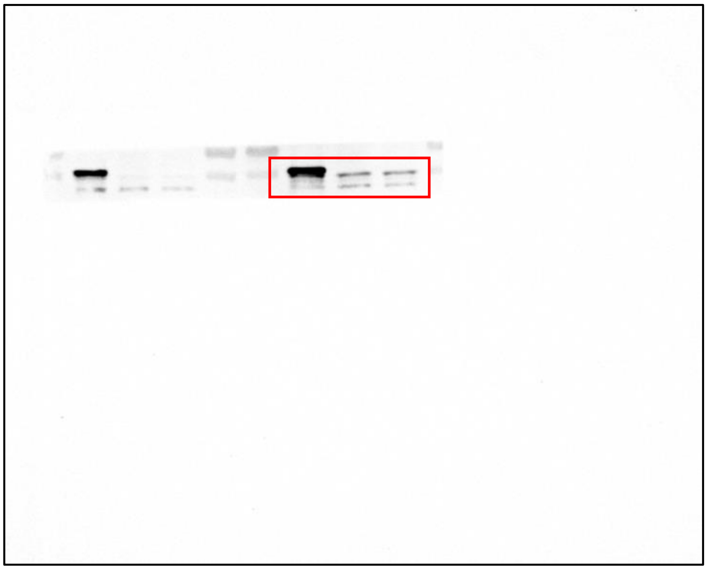

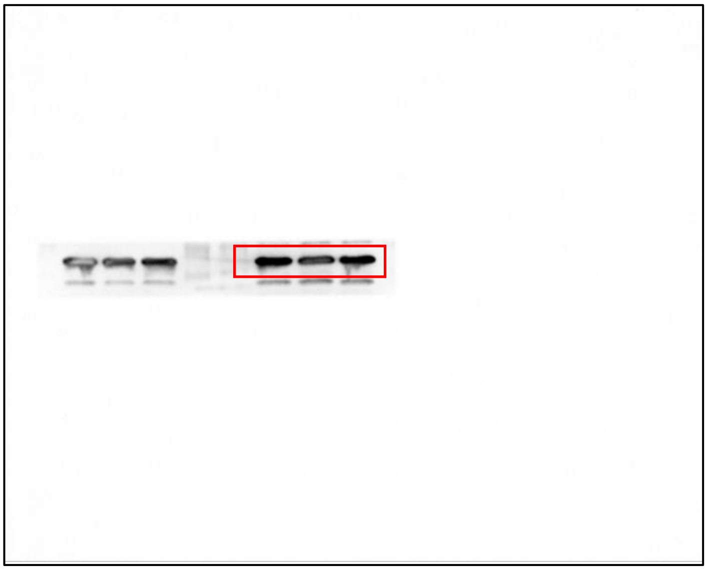

Figure 6F

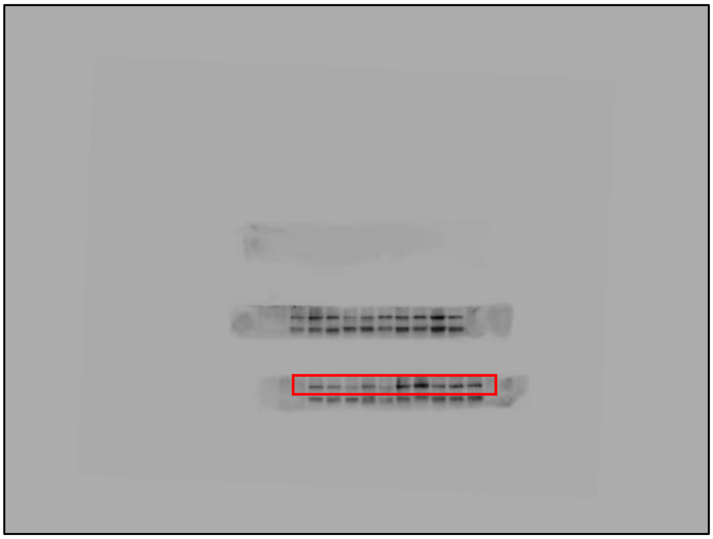

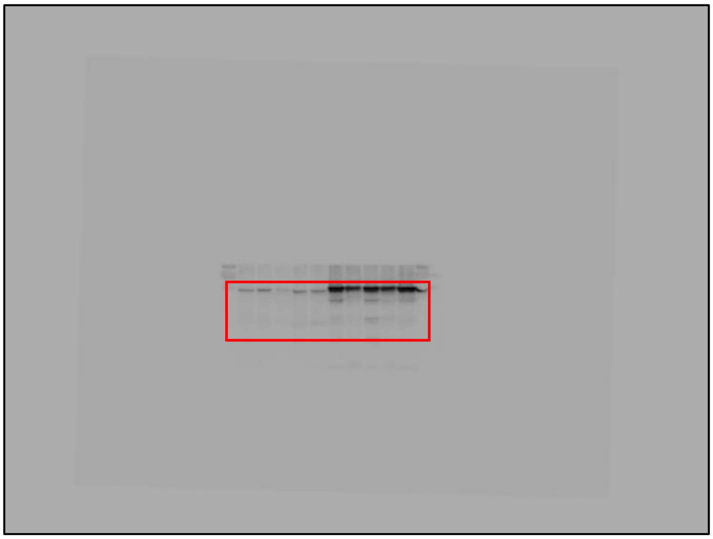

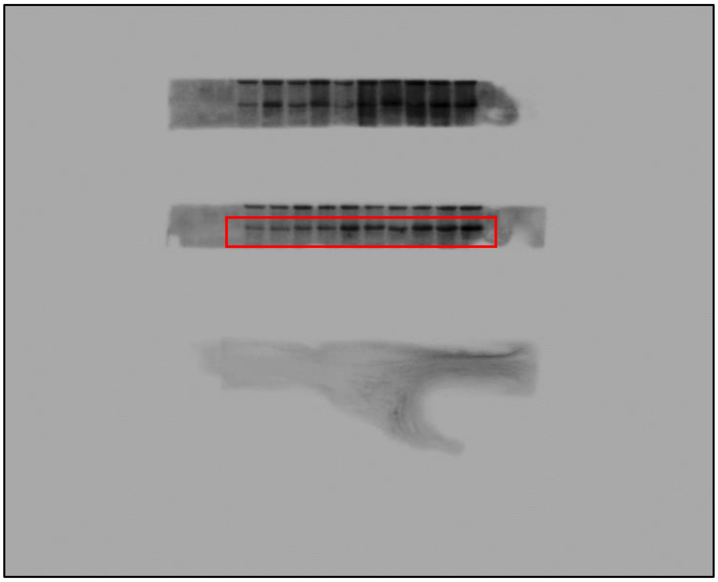

Figure S4

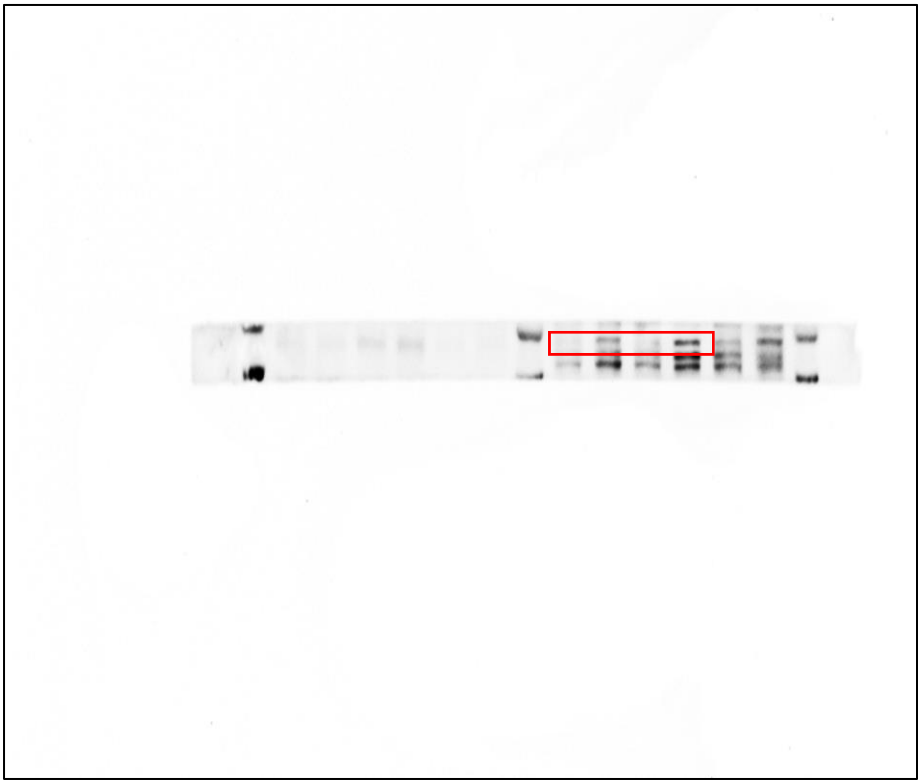

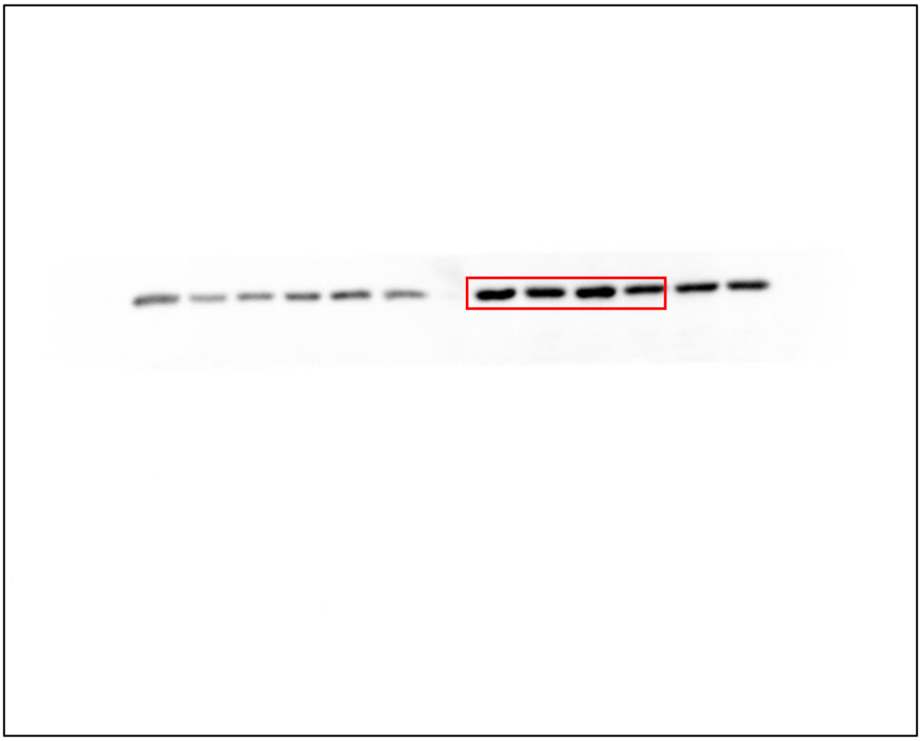

Figure S7

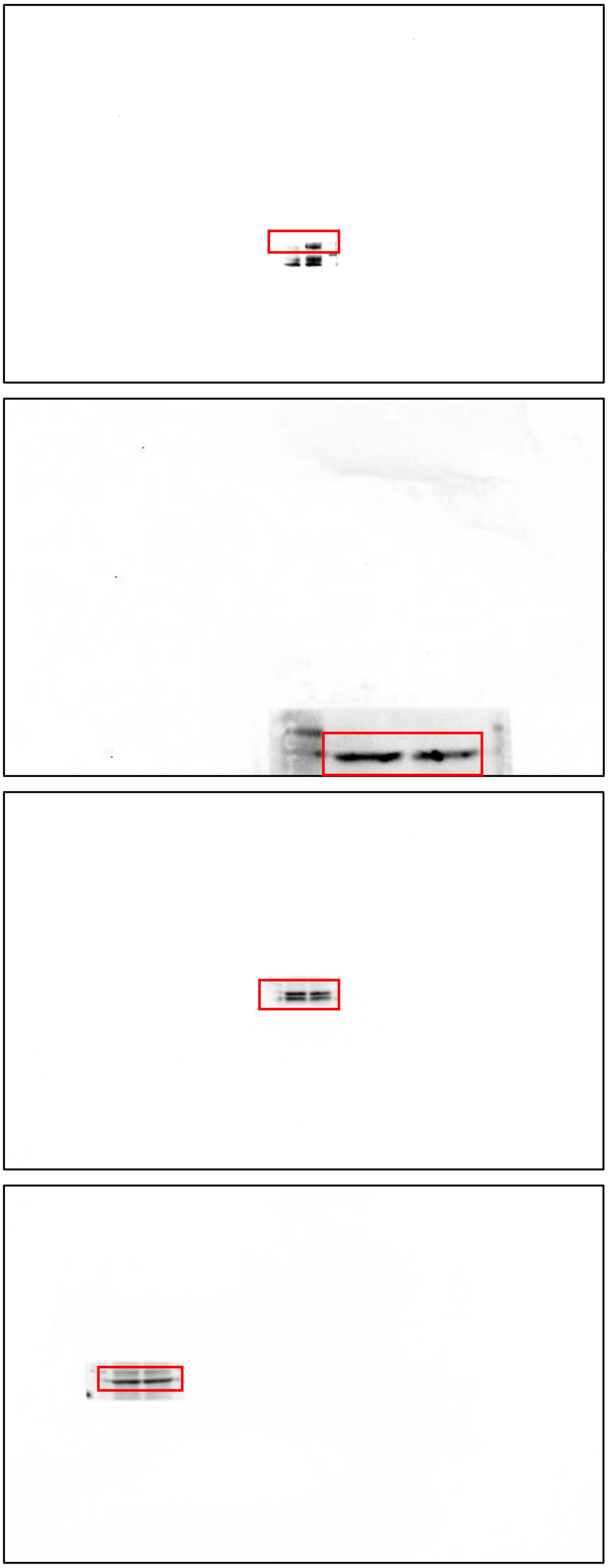

Figure S8

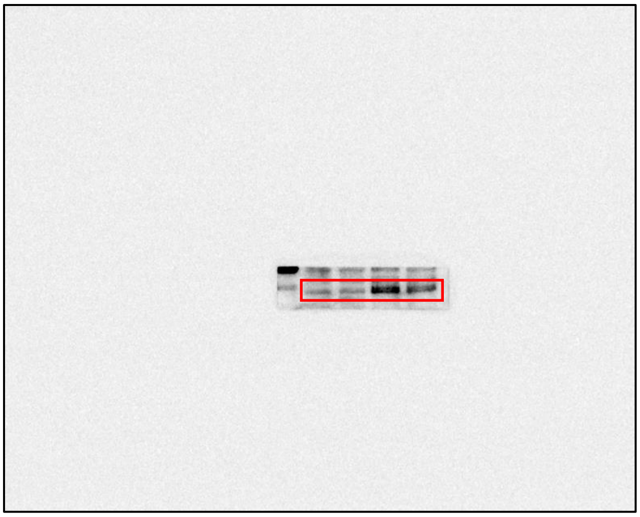

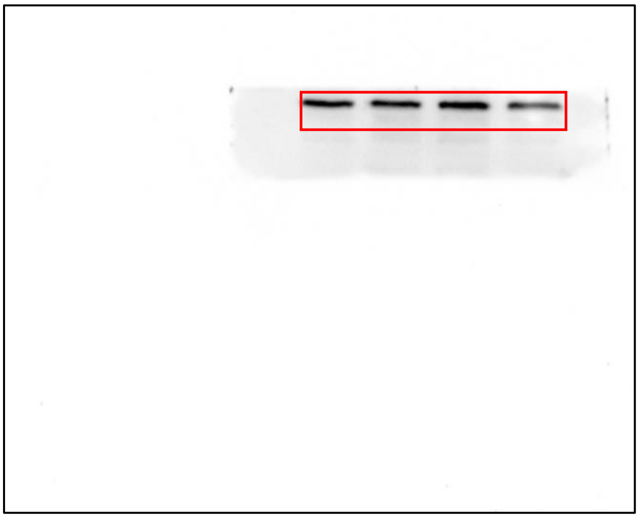

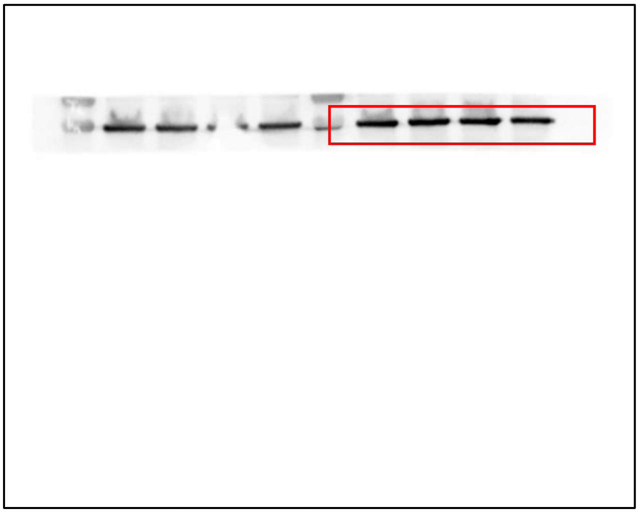

Figure S9

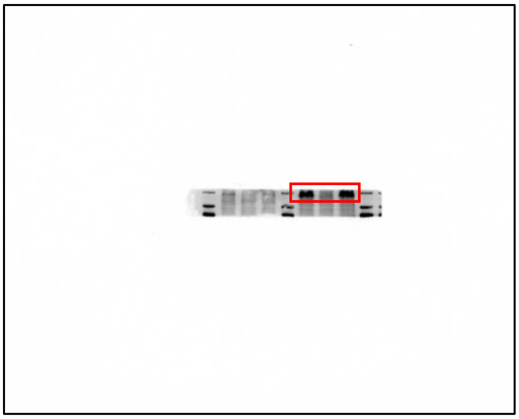

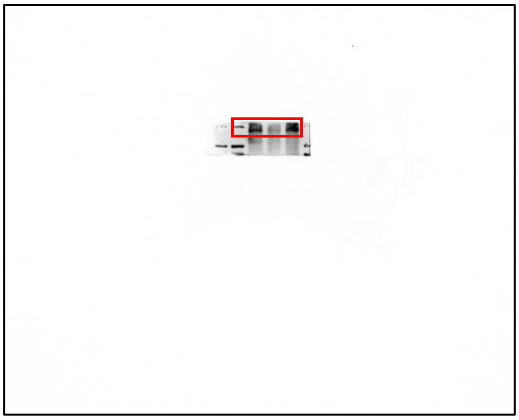

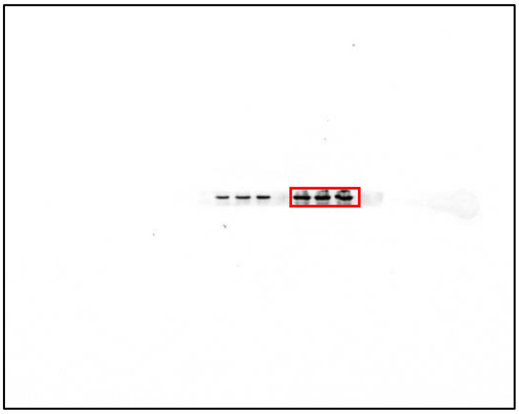

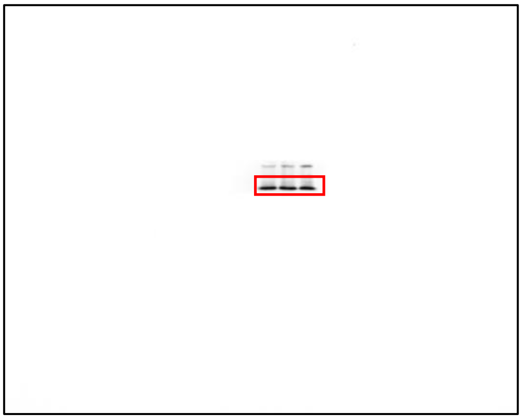

Figure S10

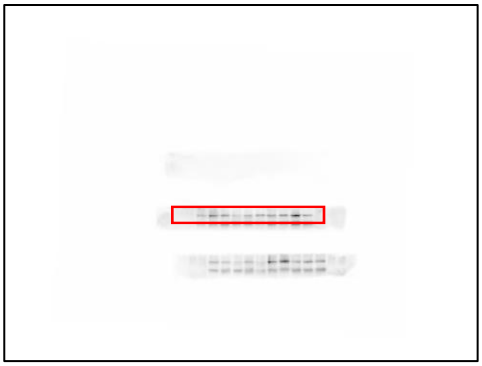

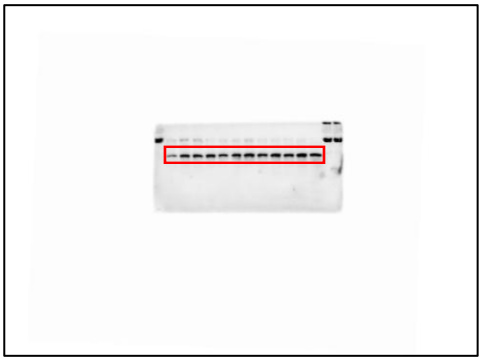

Figure S11

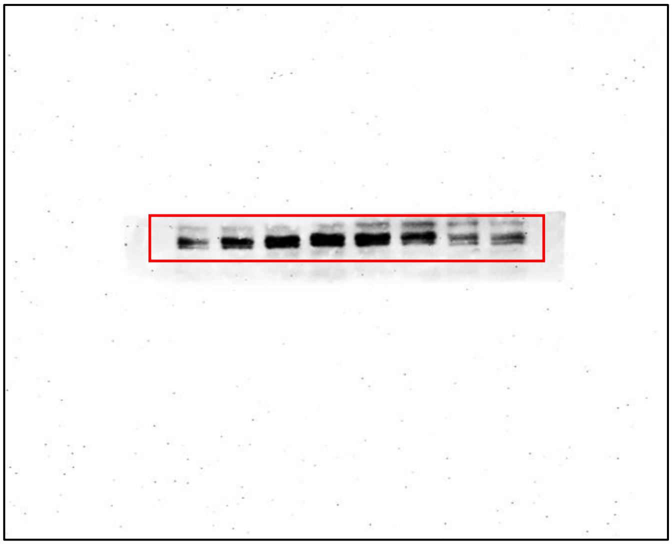

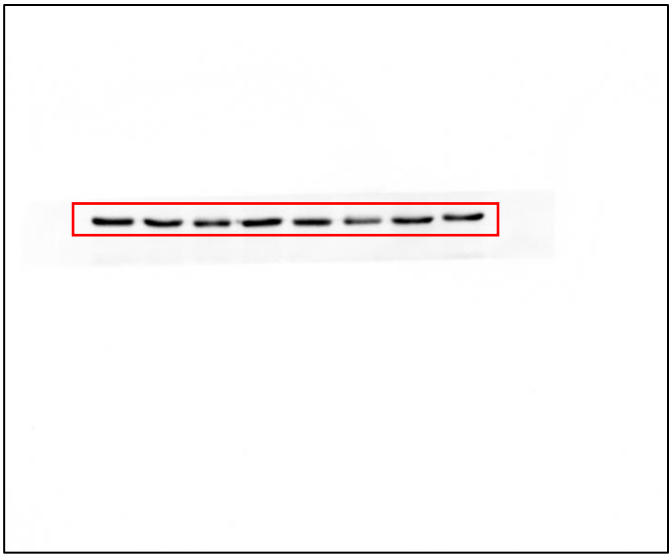

Supplement: Supplementary file 1 — Supplementary material [file 41420_2022_915_MOESM1_ESM.pdf]
